# Behavioral Timing of Interictal Spikes, But Not Rate, Correlates with Impaired Working Memory Performance

**DOI:** 10.1523/JNEUROSCI.0193-25.2025

**Published:** 2025-09-11

**Authors:** Justin D. Yi, Maryam Pasdarnavab, Laura Kueck, Gergely Tarcsay, Laura A. Ewell

**Affiliations:** ^1^Anatomy & Neurobiology, School of Medicine, University of California, Irvine, Irvine, California 92617; ^2^University of Bonn, Bonn 53113, Germany; ^3^Neurobiology of Brain and Behavior, Charlie Dunlop School of Biological Sciences, University of California, Irvine, Irvine, California 92617; ^4^Center for Learning and Memory, University of California, Irvine, Irvine, California 92617

**Keywords:** hippocampus, kainic acid, mouse, place cell, replay, temporal lobe epilepsy

## Abstract

In temporal lobe epilepsy, interictal spikes (IS)—hyper-synchronous bursts of network activity—occur at high rates in between seizures. We sought to understand the influence of IS on working memory by recording hippocampal local field potentials from male epileptic mice while they performed a delayed alternation task. Interestingly, the rate of IS during behavior did not correlate with performance. Instead, we found that IS were correlated with worse performance when they were spatially non-restricted and occurred during running. In contrast, when IS were clustered at reward locations, animals tended to perform well. A machine learning decoding approach revealed that IS at reward sites were larger than IS elsewhere on the maze, and could be classified as occurring at specific reward locations. Finally, a spiking neural network model revealed that spatially clustered IS preserved hippocampal replay, while spatially dispersed IS disrupted replay by causing over-generalization. Together, these results show that the spatial specificity of IS on the maze, but not rate, correlates with working memory deficits.

## Significance Statement

In people with epilepsy, the hippocampus can generate large electrical discharges in the period between seizures called interictal spikes (IS). Previous studies have proposed that interictal spikes cause memory impairments. We use a mouse model of epilepsy and computer simulations to study how IS impact navigation to remembered rewards. We find that when IS occur uncontrollably throughout the maze memory performance is worse, and in contrast, when they are sequestered to reward locations memory performance is better. Together our results show that interictal spikes are correlated with corrupted memory depending on when and where they occur during learning.

## Introduction

Temporal lobe epilepsy (TLE) is the most common focal epilepsy syndrome, and is often comorbid with cognitive impairments. Deficits in episodic memory and working memory are common (Helmstaedter et al., 2003; Hermann et al., 2006; Stretton and Thompson, 2012; Arski et al., 2021; Fleury et al., 2022; Caciagli et al., 2023). One clearly pathological feature of memory processing in TLE is that memory tasks promote interictal spikes (IS; Vivekananda et al., 2019), hyper-synchronous network events observed as large spikes in local field potential (LFP) recordings. The recruitment of elevated IS rates during memory tasks suggests that the network mechanisms that promote IS may be hijacking dynamics that are typically engaged by memory processes (Holmes, 2014). It is known that spatial working memory task performance is disrupted in rodent models of TLE (Naik et al., 2021), however, it is not known if the exact timing of IS with respect to different task phases (i.e., navigational epochs vs delay/memory maintenance epochs) contributes to the memory deficit as has been found in other memory models (Kleen et al., 2010; Horak et al., 2017; Quon et al., 2021; Camarillo-Rodriguez et al., 2022).

For example, as healthy animals navigate an environment, the hippocampal network is in a low synchrony state due to rhythmic inhibition (Csicsvari et al., 1999; Klausberger et al., 2003; Vandecasteele et al., 2014) in which theta oscillations orchestrate the sequential activation of individual place cells over second long time scales (Vanderwolf, 1969; Skaggs et al., 1996; Dragoi and Buzsáki, 2006; Foster and Wilson, 2007; Buzsáki and Moser, 2013). Hippocampal dis-inhibition during theta states leads to aberrant population synchrony. For example, when inhibition is reduced experimentally, theta oscillations during running can grow into large amplitude spikes that resemble bursts of IS (Boehringer et al., 2017). In epileptic rodents, IS have been reported to encroach on theta states and impact place cell spatial coding (Ewell et al., 2019).

As healthy animals consume reward or sit quietly during delay phases of the task, the hippocampus shifts to a high synchrony state and engages in brief periods of physiological network synchrony called sharp wave ripples (SWR; Buzsáki, 2015), which replay memory relevant ensembles of neurons at compressed time scales (Wilson and McNaughton, 1994; Singer and Frank, 2009; Dragoi and Tonegawa, 2011; Jadhav et al., 2012; Pfeiffer and Foster, 2013; Joo and Frank, 2018; Gillespie et al., 2021). There is evidence to suggest that there are parallels between IS and SWR neuronal dynamics and that, in principle, they could partially fulfill analogous functional roles. For example, both recruit ensembles of CA1 pyramidal cells, are accompanied by brief fast oscillations measured in cell layer LFP [pathological high frequency oscillations (HFOs) for IS and ripple oscillations for SWR], and coordinate with the cortex via sleep spindles (Bragin et al., 2007; Gelinas et al., 2016; Dahal et al., 2019; Ewell et al., 2019). Furthermore, in epilepsy, when IS rates go up during a memory task, SWR rates go down (Henin et al., 2021), indicating that IS may actually replace SWR. It is not known whether, like SWR, IS can support diverse roles in memory tasks, such as amplifying salient cues, remote replay of past animal positions and rewards, or preplay of future trajectories (Singer and Frank, 2009; Dragoi and Tonegawa, 2011; Jadhav et al., 2012; Pfeiffer and Foster, 2013; Joo and Frank, 2018; Gillespie et al., 2021).

We conduct in vivo electrophysiology and behavioral analysis in freely moving epileptic mice to test the hypothesis that IS may create spatial memory interference by promoting aberrant population level synchrony during theta states. Furthermore, we use machine learning analysis of IS recordings and build a spiking neural network model to assess the plausibility that IS may maintain reward-related working memory representations during offline states.

## Materials and Methods

### Experimental model and study participant details

#### Subjects

All experimental procedures were performed as approved by the Institutional Animal Care and Use Committee at the University of California, Irvine and according to National Institutes of Health and institutional guidelines or following European (2010/63/EU) and federal law (TierSchG, TierSchVersV) on animal care and use and approved by the county of North Rhine-Westphalia (81-02.04.2018.A006/2–mittel). All the experiments were performed using male C57BL/6 mice (Charles River). All mice were single housed under a 12 h light-/dark-cycle, in at 22 ± 2°C and humidity 55 ± 10%. Food and water were available ad libitum except for during the working memory task period when mice were either food restricted to maintain 85% of their initial weight or given a 2% citric acid water replacement when a sugar water reward was given. All efforts were made to minimize pain and reduce the number of animals used.

### Method details

#### Kainate induction of chronic TLE

Kainate injections were performed in 3-month-old C57BL/6 male mice. In one laboratory, mice were anesthetized with an intraperitoneal injection (0.1 ml/10 g body weight) of Ketamine (0.1 ml of 1 g/ml; Bela-Pharm GmbH & Co. KG), Dormitor (0.1 ml of 1 mg/ml Medetomidine hydrochloride; Orion Pharma) and Sodium chloride (0.8 ml of 0.9%; Fresenius Kabi Deutschland). Analgesia (5 mg/kg of Gabrilen, Ketoprofen) was given subcutaneously 30 min before the surgery, and Xylocaine (AstraZeneca) was used for local anesthesia. In the other laboratory, anesthesia was induced with 3–4% isoflurane and maintained at 1–2% isoflurane. Lidocaine (2 mg/kg Patterson Veterinary Supply) was used for local anesthesia. Baytril (0.5 mg, bacon flavored tablet, Bio-Serv) was used for post-operative antibiotics 5 d post-op.

Stereotactic injections were performed using a stereotactic frame (Kopf) and a microprocessor-controlled minipump (World Precision Instruments). Seventy nanoliters of 20 mM Kainate Acid (Tocris Bioscience) or saline was injected unilaterally into cortex above right hippocampus (*M*/*L* = 1.5 mm; *A*/*P* = 1.9 mm; *D*/*V* = 1.1 mm from skull surface at bregma) using a 10 ml Nanofil syringe (WPI). For animals anesthetized with Ketamine, after suturing, the antagonist Antisedan (5 mg/ml Atipamezole hydrochloride (Orion Pharma) was injected intraperitoneally (0.1 ml/10 g body weight). The incision was covered with an Antibiotic-Cream, Refobacin (1 mg/g Gentamicin) or Neosporin First Aid antibiotic-cream. Immediately after surgery we gave 1 ml of a 5% Glucosteril solution subcutaneously. Four hours after surgery, status epilepticus was terminated using diazepam (10 mg /2 ml, Ratiopharm) injected subcutaneously (0.1 ml/20 g body weight), or lorazepam (7.5 mg/kg, MWI Veterinary supply) injected subcutaneously. Ketoprofen or carprofen (5 mg/kg, Rimadyl, MWI Veterinary supply) was also injected subcutaneously on the three following days to mitigate pain. Animals were left to rest for at least 1 week before starting handling.

Kainic acid (KA, Tocris Bioscience, ItemNo: 0222) was prepared by combining 50 mg of KA powder with a 40 mM Sodium hydroxylate solution to get a stock solution of 40 mM Kainate. Aliquots were stored at −20°C and mixed 1:1 with 0.9% NaCl solution to obtain 20 mM KA.

#### Tetrode recording

Double bundle microdrives (Axona) comprised two bundles of four tetrodes separated by 3 mm to target bilateral hippocampus. The tetrodes were made of tungsten wire (Tungsten 99.95%, California Fine Wire Company) and plated with a gold solution to have impedance ∼200 kΩ. To implant the microdrives, mice were injected with the analgesic buprenorphine (0.05 mg/kg body weight) and ketoprofen (5 mg/kg body weight) to reduce pain. Twenty minutes later, mice were anesthetized initially with 3–4% isoflurane using an oxygen/air mixture (25/75%), placed on a regulated heating plate (TCAT-2LV, Physitemp) to retain the body temperature at 37°C, and head-fixed in a stereotactic frame. Anesthesia was performed via a mask with isoflurane 1–2% at a gas flow of about 0.5 ml/min. After removing the skin and other tissues from the skull, a layer of Optibond (OptibondTM 3FL, KERR) was applied. Reference and ground screws were placed anterior to the bregma. Two craniotomies were drilled for tetrode implantation bilaterally (−2 mm AP, ±1.5 mm ML) with a dental drill. After removing the dura, tetrodes were placed in 70% ethanol for 2 min before being implanted in the cortex above the hippocampus (∼0.6 mm DV). After placing the tetrodes, they were covered with heated gelatinous paraffin to protect them from the dental cement. Paraffin was made with 40 g of solid wax and 50 ml oil that were mixed at $100^{\circ }$C. The microdrive was fixed in place using dental cement (Paladur powder and liquid, Kulzer). Mice were injected with glucose monohydrate (Glucosteril, Fresenius Kabi Deutschland; injection volume 0.25 ml, s.c.) and were kept single housed on a heat-pad. They were carefully monitored twice daily and injected with the analgesic ketoprofen (5 mg/kg) to reduce pain on the following four days. One week after implantation, LFP recordings were acquired using a Neuralynx system (Digital Lynx 4SX, Sample Rates 32 kHz, filtering 1–8,000 Hz) and Cheetah 6.4.1.

Over several weeks, tetrodes were turned to the following configuration. On each side, one tetrode was positioned in the cortex for reference, complemented by three tetrodes in left and right hippocampus spanning CA1 to the dentate gyrus.

### Linear probe recording

Four weeks after Kainate injection, a high-density linear silicon probe (Neuronexus, H64LP A1×64-Edge layout, 64 channels, and 20 μm spacing) was implanted in the cortex above the right hippocampus (AP −1.9 mm, ML +1.6 mm, and DV 0.8 mm). Anesthesia and post-operative care was done as for KA injections described above with the exception including dexamethasone (MWI Veterinary Supply, 2–4 mg/kg, i.p.) during implantation and buprenorphine (MWI Veterinary Supply, 0.05 mg/kg, s.c.) and carprofen (5 mg/kg, Rimadyl, MWI Veterinary supply) was used for peri-operative analgesia. After the mouse recovered for 1 week, the probe was lowered manually over 5 d using a microdrive (3D Neuro–R2Drive, Vöröslakos et al., 2021) to a depth of approximately 2.4 mm. During all recording sessions, the probe was connected to an OpenEphys (OE) Acquisition Board via a 64-channel Intan Omnetics headstage. The signal was recorded using a custom Bonsai workflow, where the OE board output was recorded using an Intan Rhd2000 Evaluation Board Node sampled at 30 kHz (Tarcsay et al., 2022).

### Spatial alternation task

Memory task training started two weeks after KA induction. The maze apparatus is a Figure 8 shape (dimension 80 × 90 cm). Mice were trained to perform spatial alternation to receive sugar pellet rewards (200 mg, Test Diet). Mice were food restricted to 85% of their baseline weight. Training consisted of three phases: (1) habituation, (2) forced alternation, and (3) free alternation. During habituation the mouse freely explored the maze that was covered with nine sugar pallets (3 per arm). The Habituation phase was continued daily until the mouse ate all pellets in under 5 min. During the forced alternation phase mice were guided to alternate between right and left side of the maze using barriers placed on the maze by the experimenter. During the free alternation phase, the mouse was allowed to freely choose between visiting the two sides of the maze and only visits the opposite arm from the previous trial were rewarded with a sugar pellet and considered “correct.” Mice reached training criteria when they performed >80% correct choices on 2/3 consecutive sessions of the free alteration phase. During the three phases of training, there was never a delay between trials. After reaching criteria, food restriction was terminated. The mice ate freely and rested for 5–7 d before microdrive implantation was performed. After surgery, mice were retrained to run with cables and again reached criteria before being passed to the memory testing phase. Testing comprised 5 d where mice ran 15–30 trials with delays of 30 s between trials. Before and after behavioral sessions, mice were placed in a monitoring chamber (glass bowl) where they were video-LFP monitored for at least 3 h per day.

One mouse (implanted with a silicon probe) was implanted prior to any training and was run in an automated version of Figure 8 maze (48 × 48 cm). One day prior to habituation, the mouse was placed on a 2% citric acid water regiment (Urai et al., 2021). Video tracking was controlled by a Bonsai workflow and maze doors and reward ports (Sanworks, Mouse Port Assembly) were operated by an Arduino micro-controller which interfaced with Bonsai. When the mouse broke an IR beam to drink, approximately 10 μl of 5% sucrose water was dispensed as a reward. The training schedule was similar to that for the mice run on the non-automated maze, but involved habituation to the maze and automatic doors rather than eating food pellets.

On all behavior days, the mouse rested in a home cage immediately before and after the maze session for ∼15 min, during which the LFP was recorded. After the completion of all behavioral days, the mouse was video monitored in a home cage once for 14 h overnight (6:30 P.M. to 7:30 A.M.) to estimate seizure burden.

### Quantification and statistical analysis

#### IS detector

All signal processing was done in MATLAB (R2024a and R2024b, The Mathworks). Single channel LFP signals were selected based on their location being in the hippocampus (confirmed by histology) and on the amplitude of IS. The LFP was down-sampled to 1,000 Hz. The sign of the signal’s skew was estimated and used to ensure IS were oriented positively regardless of the original polarity of the signal. Then, the signals were band-pass filtered (Table 1), and peaks with a minimum prominence above a tuned threshold (Table 1) were counted as the location of IS.

**Table 1.** Interictal and ictal spike detection parameters for each animal

Animal ID	Type	Threshold (μV)	Low passband (Hz)	High passband (Hz)	*F*_1/2_ Score	Precision (%)	Recall (%)
m1	Tetrode	900	2	400	0.45	50%	32%
m2	Tetrode	900	16	400	0.92	97%	76%
m3	Tetrode	1,900	1	400	0.86	90%	71%
m4	Tetrode	1,300	1	400	0.58	69%	35%
m5	Tetrode	1,100	1	400	0.99	99%	99%
m6	Tetrode	700	1	400	0.78	83%	62%
m7	Probe	1,700	1	400	0.70	75%	56%

Settings were optimized according to the procedure detailed in the Methods.

#### Detector tuning

For each animal, random 3-min segments were selected from representative three behavioral and one sleep sessions for a total of 12 min per animal. Windows around interictal and/or ictal spikes were labeled manually using the MATLAB SignalLabeler GUI and used as a “ground truth” for tuning the detector. One trained author (J.D.Y.) manually labeled the raw LFPs, where the sign of the LFP was flipped such the largest events faced negatively. IS were inspected visually for large negative deflections that strongly deviated from baseline. The start and end of the window were defined as when the envelope of the IS amplitude returned to the baseline level by visual inspection. Under this manual method, the window range had a mean = 100 ms, standard deviation = 92 ms, a minimum = 14 ms, max = 724 ms. The detector was run on this ground truth dataset and the threshold, low- and high-pass bands were varied to maximize the *F*_1/2_ score for each animal (Table 1). True positives (TP) were counted if the detector labeled exactly one spike within the labeled window. False positives (FPs) were either (1) any additional spikes within a labeled window or (2) any spike outside a window. False negatives (FNs) were windows that contained no detected spike. True negatives were not evaluated. These values were used to calculate the Precision, Recall, and *F*_1/2_ score using the following equations:

$$\mathrm{Precision}=\frac{\mathrm{TP}}{\mathrm{TP}+\mathrm{FP}},\;(1)$$

$$\mathrm{Recall}=\frac{\mathrm{TP}}{\mathrm{TP}+\mathrm{FN}},\;(2)$$

$$F_{\beta }=(1+\beta^{2})\frac{\mathrm{Precision}\times \mathrm{Recall}}{(\beta^{2}\times \mathrm{Precision})+\mathrm{Recall}}.\;(3)$$

The *F*_1/2_ score was chosen to favor Precision roughly twice as much as Recall (*β* = 1/2; i.e., only selecting events that are very high above the noise floor) to avoid including non-interictal/ictal spike noise contaminating the data.

#### Binning maze zones

For a given session, the trajectory of the animal was plotted and segmented into zones. Trajectories across sessions were aligned and binned into 4×4 cm bins. Each trajectory was fit by a rectangle and a dissecting line after calculating the coordinates of the four corners and the center of the maze. Coordinates were used to break the maze into zones with user-defined size including delay (40 cm of central arm), stem and choice (15×15 cm), outer arm, and reward zones (15×25 cm). To perform trial-wise analyses, the session was parsed into individual trials based on the sequence of entering the zones. For the automated maze, spurious “positions” that were outside the maze due to tracking errors were removed manually by inspection post hoc.

#### Spatial information of interictal activity

To get a sense of the “spread” of spikes on the maze, we treated the IS as if they were generated from a single “place cell” and applied spatial information analysis to its activity (Skaggs et al., 1992). First, the maze was binned into a 15×15 grid, and the occupancy and number of spikes was calculated to get rates, *λ*_*i*_, and occupancy probabilities, *P*(*x*_*i*_). These were used in the information rate (bits/s) formula provided by Skaggs et al. (1992):

$$I=\sum_{i}\lambda_{i}P(x_{i})\log_{2}\;\frac{\lambda_{i}}{\sum_{i}\lambda_{i}P(x_{i})},\;(4)$$

where the original integral has been replaced by a sum over occupied spatial bins, each indexed by *i*. Finally, to get information per spike (bits/spike), the quantity, $I_{\mathrm{spike}}=I/\sum_{i}\lambda_{i}P(x_{i})$, was computed. This quantity was computed for each session.

To study how locomotion impacts *I*_spike_, for each brief rhythmic interictal discharge (BIRD), *i*, we computed the distance traveled as:

$$d_{i}=\sum_{j}\|{\vec{x}}_{ij}-{\vec{x}}_{i(j-1)}{\|}_{2},\;(5)$$

which is the sum of distances along the path defined by successive spikes indexed by *j* (compare to simple displacement from the position at the start and end of the BIRD). The operator || · ||_2_ is the Euclidean norm. A generalized linear model with mixed effects (GLME) was then fit, with specification:

$$I_{\mathrm{spike}}^{k}∼1+\langle d_{k}\rangle +(1|\mathrm{animal}),\;(6)$$

where the index *k* is a per-session index, and 〈*d*_*k*_〉 is the mean BIRD distance traveled within that session. The model was fit in MATLAB using the fitglme() function and Gamma distribution with reciprocal link. Gamma regression was selected since $I_{\mathrm{spike}}^{k}\in [0,\infty )$ and diverged from a normal distribution when inspected on a quantile-quantile plot. For each session, the alternation task performance was also fit to compare the spatial information to performance as:

$$\mathrm{Performance}∼1+I_{\mathrm{spike}}^{k}.\;(7)$$

The MATLAB fitglm() function was used to fit the regression and dispersion was estimated from the data.

#### Zone-specific IS rate analysis

Mouse location was binned into zones of the maze: “Delay,” “Choice,” “Reward,” and “Outer Arm” regions of interest. For each zone, the observed spike counts were calculated by calculating the number of IS in a given zone. These observed counts were compared to expected counts which were calculated by multiplying the percent of time in each zone by the total spike count. Observed and expected spike counts were compared with a *χ*^2^ test.

To estimate the zone-specific influence on the observed IS number of spikes for a given animal in each zone, *S*_*z*,*a*_, we employed a Bayesian approach to infer zone-specific “gains,” *η*_*z*_, which were applied to an animal-specific “baseline” IS rate, *ρ*_*a*_ as:

$$S_{a,z}∼\mathrm{Poisson}(T_{z,a}\rho_{a}\eta_{z}),\;(8)$$

where *T*_*z*,*a*_ is the number of seconds in the zone *z* spent by animal *a*. [Note the correspondence of this parametrization to that of a standard generalized linear model (GLM) with a Poisson distribution and log link function via the identity, $e^{\sum_{i}\beta_{i}x_{i}}=\Pi_{i}e^{\beta_{i}x_{i}}$, where *β*_*i*_ and *x*_*i*_ are generic regression coefficients and predictors.] The parameters to be estimated had priors of the following form:

$$\rho_{a}∼p_{a}=\mathrm{LogNormal}(-1,0.3),\;(9)$$

$$\eta_{z}∼p(\eta_{z})=\mathrm{LogNormal}(0,1).\;(10)$$

Therefore, the posterior distribution was expressed as

$$\begin{array}{rl} \;p(\theta |S_{z,a}) & \propto p(S_{z,a}|\theta )p(\theta )\\ & =\mathrm{Poisson}(T_{z,a}\rho_{a}\eta_{z})p(\rho_{a})p(\eta_{z}). \end{array}\;(11)$$

The model was specified in the probabilistic programming language Turing.jl in Julia (version 1.10.2, Bezanson et al., 2014), with packages managed with DrWatson.jl (Datseris et al., 2020). Four independent chains, each run for 1,000 iterations with 500 warm-up samples, were run using the No-U Turns Sampler (NUTS, Hoffman and Gelman, 2011) with a target acceptance ratio of 65% to estimate a posterior distribution for the parameters. $\hat{R}$ values and effective sample sizes were checked to ensure convergence, mixing, and sampling efficacy of the Monte Carlo Markov chains.

Ninety-five percent credible intervals (1 − *α*) were estimated for each parameter by using the highest posterior density (HPD) method. The credible interval for each *η*_*z*_ was compared to a “null” value of 1, and for those which did not overlap with 1, a “significance level” was estimated by lowering the (HPD) threshold *α* until the credible interval contained 1.

To assess the model fit, samples from the posterior predictive distribution were taken and used to generate “replicates” of the data, $S_{i}^{\mathrm{rep}.}$ (Gelman et al., 2013). The distribution of means of the replicates were compared to each observed data point for agreement. As a check of model specification sensitivity, mean squared errors were calculated for this model using the replicates and observed data, and then compared against a “clamped” model fit where *η*_*z*_ = 1 for all zones. The two models had Akaike information criterion values of approximately 1.30 × 10^4^ for the full model versus 1.48 × 10^4^ for the clamped model. Thus, the full model was retained for its interpretability and improved prediction performance.

#### IS LFP embedding and classification

For each IS that occurred on the maze, the single channel LFP signal was extracted ±100 ms from the detection time. Then, the LFP was down-sampled to 2,000 Hz and transformed to a z-score. The LFPs for each animal on all delayed alternation behavior sessions were then non-linearly embedded with *t*-SNE (with default parameters) to get a 2-D feature vector. A bagging ensemble of trees (Breiman, 1996, 2001) was fit using MATLAB with fivefold cross-validation (including stratification into groups with similar proportions of each discrete class) to classify whether or not the IS occurred in either reward zone based on the feature vector’s position in 2-D space. The area-under-the-curve (AUC) of the receiver operating characteristic (ROC) curve was computed and compared to the animals mean performance over five sessions using standard linear regression. Note that the qualitative results did not change when the classifier was trained on the full-dimensional LFP waveforms instead of the *t*-SNE embedding, suggesting the embedding faithfully reduces the dimensionality by persevering relevant features.

To compare the amplitudes of the IS events under different conditions, the root-mean-squared (rms) amplitude was computed for each IS. To compare across animals, the raw rms values were divided by the standard deviation of the rms for all the IS of a given animal. Two-sample *t*-tests were used to compare the distributions of amplitudes between IS inside versus outside reward zones, and IS at reward during correct versus incorrect trials.

#### Inferring trial-to-trial behavioral state from task performance

The efficacy of decision-making depends in part on the underlying behavioral state of the animal, e.g., whether the animal is engaged with the task or has a lapse in performance. This dependency of task performance and neural dynamics on a latent behavioral state has been modeled using models that capture auto-regressive dependencies across trials (Escola et al., 2011; Fründ et al., 2014; Lueckmann et al., 2018; Ashwood et al., 2022).

Borrowing ideas from Ashwood et al. (2022), we modeled the trial-to-trial performance using a hidden Markov model (HMM) with states inferred from the data as follows. Consider discrete states indexed as *s* ∈ {1, 2, …, *N*}. The probability of an animal making a “correct” choice on trial *i* depends on the state as:

$$p(c_{i}=\mathrm{Correct}|s_{i})∼\mathrm{Bernoulli}(p_{s}).\;(12)$$

In other words, the performance is like flipping a biased coin with probability of “heads” *p*_*s*_. The value of *c*_*i*_ is considered as the “emission” of the hidden Markov chain. The state can change from trial-to-trial, and thus the probabilities of transitioning between different states are expressed as:

$$p(s_{i}|s_{i-1}^{\prime })∼a_{ss^{\prime }},\;(13)$$

where $a_{ss^{\prime }}$ is an entry in the transition matrix $A\in R^{N\times N}$. The initial state on the first trial is drawn from:

$$p(s_{0})∼\mathrm{Categorical}(\alpha_{0}),\;(14)$$

where $\alpha_{0}\in R^{N}$ is the probability of initializing in each of the N states. These parameters were initialized as:

$$A_{\mathrm{init}.}=\frac{1+\epsilon }{1+N\epsilon }I^{N\times N},\;(15)$$

where $I^{N\times N}$ is the identity matrix, ϵ=0.5 is a parameter to control the relative strength of transitions between states versus persisting within the same state, the initial state as:

$$\alpha_{0}=[1/N,\ldots ,1/N{]}^{⊤},\;(16)$$

and finally, each *p*_*s*_ took one of *N* uniformly spaced values from 0.1 to 0.9.

To train the HMM, all of the choice data for each trial from each epileptic animal was concatenated into a vector and the end of each session was noted. Then, the Baum–Welch expectation-maximization procedure was applied to this concatenated vector (re-initializing when a session ended) to find the optimal values of the initial state distribution, the transition matrix, and the emission probabilities for each state (Rabiner, 1989). Using the optimized HMM parameters, the most likely state sequence given the observed choice data was computed using the Viterbi algorithm. Marginal probabilities of each state were also estimated using the forward-backwards scheme. HMM algorithms were used from the HiddenMarkovModels.jl software package in Julia (Dalle, 2024). This procedure was conducted for *N* = 2 and *N* = 3. The two HMMs had similar log-likelihoods after Baum–Welch estimation (−324.8 and −323.5 respectively), and so only the *N* = 3 case was retained for further analysis. Finally, the hierarchical bootstrap method (Saravanan et al., 2020) was applied to estimate delay period exit times by stratifying the data into three states, then sampling with replacement a single trial from an animal weighted by the number of trials that animal had within that state until the a sample of the same size as the original data in each state was generated. The mean of these samples was computed for 1,000 replicas.

#### Inferring behavioral state-dependent IS activity in the delay zones

The inferred marginal probabilities of each state sequence from the forward-backward algorithm, *p*(*s*_*i*_), were used as a prior to parameterize a variant of the firing rate model. The model likelihood was specified as:

$$\begin{array}{rl} \;p(S_{i}|s_{i};\rho_{a}) & ∼\mathrm{Poisson}(T_{\mathrm{delay},i}\rho_{a}\Sigma_{i=1}^{N}\eta_{s_{i}}p(s_{i})),\\ \rho_{a}∼p(\rho_{a}) & =\;i.i.d.\max(\mathrm{Normal}(0.5,0.5),0),\\ \eta_{s_{i}}∼p(\eta_{s_{i}}) & =i.i.d.\mathrm{LogNormal}(0,1). \end{array}\;(17)$$

The variable *S*_*i*_ is the number of spikes in the delay period on trial *i*. The term $\sum_{i=1}^{N}\eta_{i}p(s_{i})$ is the sum of gain terms $\eta_{s_{i}}$ each weighted by the marginal probability of being in state *s*_*i*_ ∼ *p*(*s*_*i*_). *T*_delay,*i*_ was defined as the time in seconds the animal spent in the delay zone at the start of the trial *i*. This model can be interpreted as applying a state-specific scalar gain $\eta_{s_{i}}$ to an underlying animal-specific firing rate *ρ*_*a*_. The prior for *ρ*_*a*_ was chosen to be weakly informative of the fact that the previous Bayesian model returned animal-specific mean rates centered around 0.5 Hz, truncated at zero to exclude negative rates. Modifying the standard deviation of this prior from 0.5 to [0.1, 0.8] had no effect on the qualitative conclusions of the inferences. The model was again estimated in Turing.jl using 5,000 samples from the NUTS sampler, initialized as before.

To validate the model estimated gains $\eta_{s_{i}}$, the discrete state sequence from the Viterbi algorithm was used to group spike counts into *N* distributions. A Kruskal–Wallis test was used to compare the spike count distributions. As posterior predictive checks, the means of the posterior mean *ρ*_*a*_ values were compared to the observed mean rate of IS from the data. Also, the distribution of predicted marginal mean spike counts $E(S_{i}^{\mathrm{rep}.})$ and the distribution of marginal means counts conditioned on the Viterbi-estimated state, $E(S_{i}^{\mathrm{rep}.}|s_{i})$, were compared to their point-estimates from the observed data, $E(S_{i})$ and $E(S_{i}|s_{i})$. Note that $S_{i}^{\mathrm{rep}.}$ denotes samples from the posterior predictive distribution (Gelman et al., 2013).

#### In silico model of IS and place coding replay

##### Spiking model of hippocampal replay

To isolate the effects of IS on hippocampal coding required for behavioral navigation, we modified the spiking neural network model of place cell replay in CA3 described by Ecker et al. (2022) and then updated by Liu et al. (2023) to include CA1. Pyramidal cells (pyr., *n* = 1,250 region) and interneurons (int., *n* = 100 per region) in CA1 and CA3 were modeled using the adaptive exponential leaky integrate-and-fire (AELIF, Brette and Gerstner, 2005) model:

$$\begin{array}{rl} C_{m}\frac{dV}{dt} & =-\left(g_{L}\left(V\left(t\right)-E_{L}\right)-g_{L}\Delta Te^{\frac{V\left(t\right)-\theta }{\Delta T}}+I_{s}\left(t\right)+w\left(t\right)\right),\\ \tau_{w}\frac{dw}{dt} & =a\left(V\left(t\right)-E_{L}\right)-w(t),\\ & \text{if spike},w\leftarrow w+b,\\ I_{s}\left(t\right) & =g_{\mathrm{AMPA}}\left(t\right)\left(V-E_{\mathrm{AMPA}}\right)+g_{\mathrm{GABA}}(t)(V-E_{\mathrm{GABA}}), \end{array}\;(18)$$

where *V*(*t*) is the membrane potential, *w*(*t*) is an adaptation current, and *I*_*s*_(*t*) are the sum of synaptic currents. Furthermore, *C*_*m*_ is the membrane potential, *g*_*L*_ and *E*_*L*_ are the leak current conductance and reversal potential, *θ* is the spike threshold, Δ*T* is the threshold sharpness, *τ*_*w*_ is the time constant for adaptation, *a* and *b* are parameters specifying how the adaptation current evolves between and following neuron spikes, respectively (see Table 2 for values which approximately correspond to those in Ecker et al., 2022). The synaptic conductances *g*_AMPA_ and *g*_GABA_ were bi-exponential functions as in Ecker et al. (2022), with *E*_AMPA_ = 0 mV and *E*_GABA_ = −90 mV. All neural simulations were specified and run in NEST v3.7 (Espinoza Valverde et al., 2024) with Python 3.12.3.

**Table 2.** Spiking neuron parameters for model Equation 18

Parameter (units)	Value (pyr., int.)
*C*_*m*_ (pF)	180, 118
*g*_*L*_ (nS)	4.3, 7.5
*E*_*L*_ (mV)	−75, −74
*θ* (mV)	−24, −57.7
Δ*T* (mV)	4.23, 4.6
*V*_peak_ (mV) (when a “spike” is detected)	−3.25, −34.78
*V*_reset_ (mV) (reset voltage after spike)	−29.7, −65
*t*_ref._ (ms) (refractory period)	5.9,1
*τ*_*w*_ (ms)	83.4, 178.58
*a* (nS)	−0.27, 3.05
*b* (pA)	206.84, 0.91

To simulate the plasticity induced by repeated exploration of a maze environment, we adopted a modified place- and theta-modulated spike-timing-dependent plasticity (STDP) paradigm introduced in Ecker et al. (2022) and Liu et al. (2023); a similar form model was experimentally validated in Liao et al. (2024). The original model only considered a single 3 m long linear track, whereas our task involves alternating across two separate arms of a maze. Therefore, to understand whether the two arms are re-activated during replay separately, we modified the “exploration” paradigm to take place on two 150 cm arms pointed left and right, with the mouse starting at the midpoint and “teleporting” back to the midpoint once it reached either end. For 10 min, leftward and rightward trajectories were chosen at random according to a 90% chance of alternation. The simulated mouse ran at 35 cm/s with a theta oscillation frequency of $f_{\theta }=7$ Hz. In both CA1 and CA3, 30% of pyramidal cells were selected as place cells. Each was given a place field with center *x*_*i*_ drawn uniformly from the total length of the maze. During the “exploration” phase, only pyramidal cells were simulated as inhomogeneous Poisson processes with firing rates as:

$$\lambda_{i}(t)=\lambda_{\max}\left[\frac{1}{2}+\frac{1}{2}\cos\;(2\pi f\theta t+\frac{\pi }{\sigma }\mathrm{sign}(x_{i})(x(t)-x_{i})\right]e^{\frac{-(x(t)-x_{i}{)}^{2}}{2\sigma^{2}}},\;(19)$$

where the maximum firing rate at the center of the place field was $\lambda_{\max}=20$ Hz, and the width of the place field was *σ* = 7 cm. This equation encapsulates place tuning and theta phase precession (Ecker et al., 2022; Liu et al., 2023). Non-place cells fired with a mean rate of 0.1 Hz. To simulate IS which clustered at the same location of the maze during exploration (for example, the reward ports), all CA3 pyramidal cells received a pulse of spikes at $\lambda_{\max}=2,000$ Hz described according to:

$$\lambda_{\text{IS}}\left(t\right)=\lambda_{\max}\sum_{x_{\text{IS}}^{j}\in [-100\;\mathrm{cm},+100\;\mathrm{cm}]}e^{\frac{-(x(t)-x_{\text{IS}}^{j}{)}^{2}}{2\sigma^{2}}},\;(20)$$

where *σ*_IS_ = 4 cm. A single spike train was drawn from *λ*_IS_(*t*) and broad-casted to all the CA3 pyramidal cells, but each neuron only received each spike with an independent probability of 1%. During exploration the pyramidal cell spiking was clamped to *λ*_*i*_(*t*) induce sequences encoded in the weight matrix tuned by STDP (Ecker et al., 2022; Liu et al., 2023; Liao et al., 2024) . The normalized synaptic weights, *w* ∈ [0, 1], were updated according to standard STDP rules (Gütig et al., 2003) as:

$$\Delta w=\left\{ \begin{array}{cc} -\lambda \alpha w^{\mu_{-}}e^{\frac{-|\Delta t|}{\tau_{-}}} & \text{if}\;\Delta t\leq 0\\ \lambda (1-w{)}^{\mu_{+}}e^{\frac{-|\Delta t|}{\tau_{+}}} & \text{if}\;\Delta t>0, \end{array}\right.\;(21)$$

where μ_±_ = 0 (weight-independent updating rule) and Δ*t* = *t*_post_ − *t*_pre_, *λ* is the step size parameter, *α* is an asymmetric parameter controlling synaptic depression, and *τ*_+_ and *τ*_−_ are the time scales of facilitation and depression respectively. For CA3-to-CA3 pyramidal neuron synapses STDP was symmetrically facilitating as in Liu et al. (2023), thus, $\lambda =\frac{0.08}{w_{\max}}$ nS, *α* = −1, *τ*_±_ = 62.5 ms, and $w_{\max}=40$ nS. For CA3-to-CA1 synapses, $\lambda =\frac{0.8}{w_{\max}}$ nS, *α* = 0.4, *τ*_+_ = 20 ms, *τ*_−_ = 40 ms, and $w_{\max}=40$ nS. Weights were initialized as 0.3LogNormal(0,1) nS for CA3 and 0.7LogNormal(0,1) nS for CA1. Synapses were formed between CA3 pyramidal neurons recurrently and fed forward to CA1 neurons with 10% probability for each pair of cells. The training procedure above was repeated 10 times for control and epileptic conditions to generate different replicas.

To simulate spontaneous replay during “offline” states such as the delay period between trials, the full network with pyramidal cells and interneurons with AELIF dynamics was constructed. The final pyramidal-to-pyramidal cell weights learned by STDP after all exploration trials were used to parameterize static synapses. CA3 pyramidal cells were stimulated by background activity from the dentate gyrus that was assumed to have a pooled rate of 12 Hz and synaptic weight of 15 nS. The connections between all other cell types are detailed in Table 3.

**Table 3.** Synaptic parameters during “offline” state modeled after (Ecker et al., 2022)

Pre, Post	Weight (nS)	Delay (ms)	Rise time (ms)	Decay time (ms)	Prob. connection
CA3 pyr., CA3 pyr.	STDP	1	1.0	9.0	0.1
CA3 pyr., CA1 pyr.	STDP	1	1.0	9.0	0.1
CA3 pyr., CA3 int.	0.85	1	1.0	9.0	0.1
CA1 pyr., CA1 int.	0.85	1	1.0	9.0	0.1
CA3 int., CA3 pyr.	0.65	1	0.3	3.0	0.25
CA1 int., CA1 pyr.	0.65	1	0.3	3.0	0.25
CA3 int., CA3 int.	5	1	0.3	3.0	0.25
CA1 int., CA1 int.	5	1	0.3	3.0	0.25
DG, CA3 pyr.	20	1	0.65	5.4	0.25

For comparing how the IS distributions on the maze affected network cueing, we simulated a case with high spatial information where IS were given to only CA3 cells with place fields at ±100 cm (i.e., the “reference point”), and a case with low spatial information where the place cells which were stimulated varied uniformly over the interval (±0 cm, ±150 cm) on each trial. All spikes from the IS were delivered to active place cells (defined as when place cells achieved 10% of their maximum firing rate). To “cue” replay, the weight of background activity was reduced to 10 nS, and CA3 place cells associated with different zones were stimulated with a 20 ms burst of spikes sampled from a Poisson process at 30 Hz with a synaptic weight of 80 nS from the simulated dentate gyrus. Cues were given to CA3 place cells on 20 cm wide intervals centered on equally spaced (20 cm) locations between 0 and 80 cm away from a reference point. Ten independent networks were trained for 300 s, and then for each case, 10 random seeds were used to initialize simulations for each cue center.

##### Analysis of simulated LFP

The “LFP” proxy of the network was computed as the sum of all synaptic currents delivered to a random subset of 200 CA1 pyramidal cells:

$$\text{LFP}\left(t\right)=\frac{1}{4\pi \sigma r}\sum_{i}g_{i}(t)(V\left(t\right)-E_{i}),\;(22)$$

sampled at 1,000 Hz where the extracellular conductivity *σ* =0.3 S/m and the distance from each current source to the electrode was set to be *r* = 5 μm. The choice of these parameters only affects a scalar gain (Ecker et al., 2022). Replay events were detected by detecting peaks with a prominence of 1 mV and minimum distance of 200 samples on the lowpass filtered LFP at 200 Hz with a seventh order Butterworth filter. Once the peaks were found, 150 ms on either side of the peak were selected and used for further analysis. The power spectral density (PSD) of the LFP was estimated using Welch’s method with 256 samples per segment, 32 sample overlap, and 1,024 FFT points. The PSD was computed on 5 s long segments of spontaneous activity from each of the 10 replica networks. The continuous wavelet transform (CWT) was computed on the averaged replay LFP using a complex Morlet wavelet with bandwidth of 1.5 and center frequency of 1.0 at 200 logarithmically spaced frequency bands between 10^1.2^ and 10^2.5^ Hz. To compute the CWT, only one replica network was selected and the replays over 5 s were used for averaging. Signal processing was done using Scipy (Virtanen et al., 2020) and the PyWavelets package (Lee et al., 2019).

##### Analysis of simulated replay events

After replays were detected by the LFP, the place cell activity was extracted and used to reconstruct the maze position using the population vector method (Zhang et al., 1998):

$$\hat{x}[t]=\underset{x}{\text{argmax}}\;\sum_{i}\delta (x-x_{i})n_{i}[t],\;(23)$$

where *δ*(*x*) is the Dirac delta function, *x*_*i*_ is the place field center, and *n*_*i*_[*t*] indicates the number of spikes neuron *i* fired within a discrete 25 ms time bin.

## Results

### Epileptic mice exhibit persistent focal interictal activity

**Figure 1.**
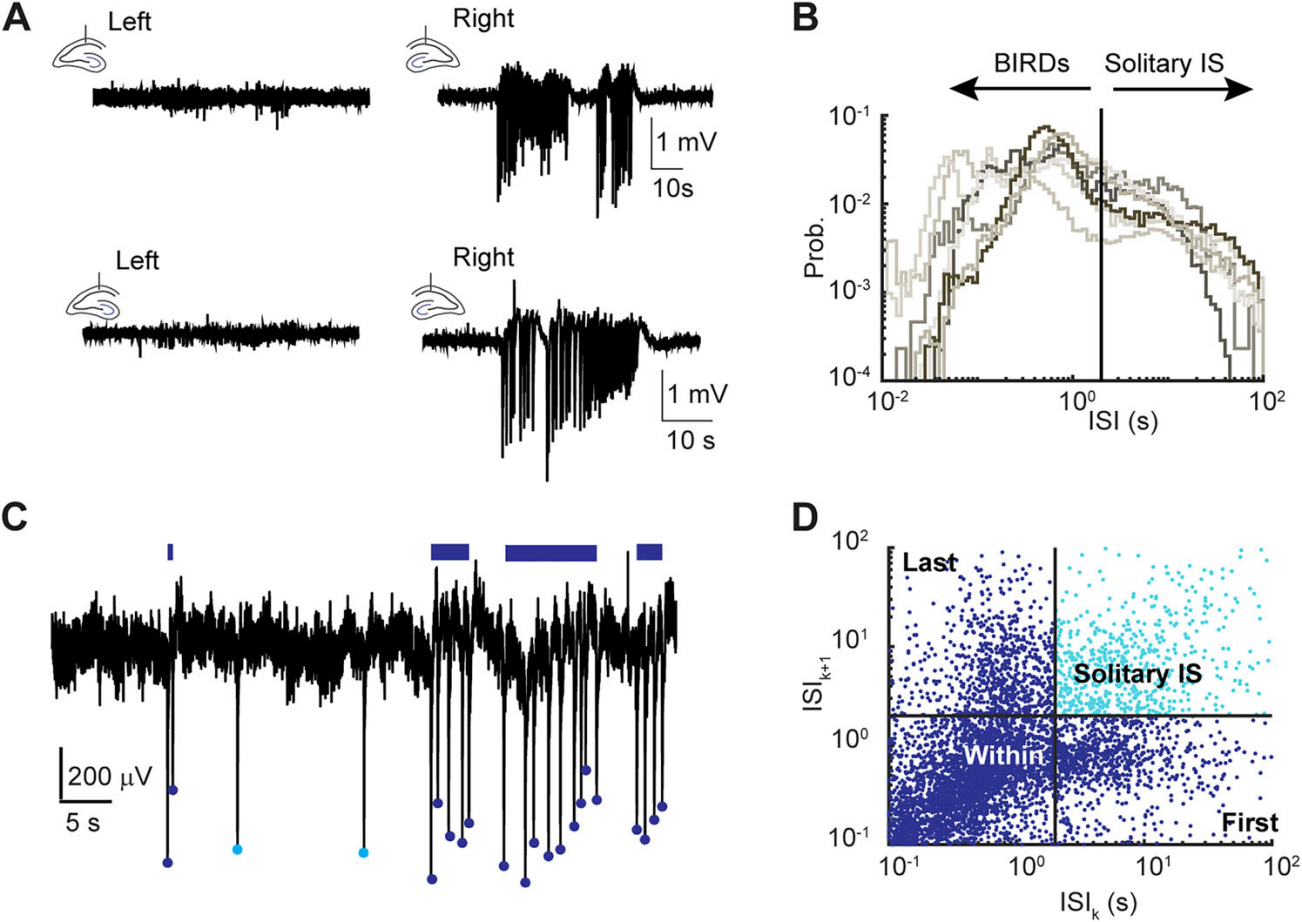
KA mice exhibit spontaneous seizures and interictal activity. ***A***, Two examples of seizures recorded from the hippocampus bilaterally where the right hemisphere was injected with KA. Spikes were detected continuously both during subclinical seizures and in the interictal period. ***B***, For each KA animal, the inter-spike intervals between each interictal spike (IS) was used to classify IS as solitary IS or chains of IS called brief rhythmic interictal discharges (BIRDs). IS with inter-spike intervals greater than 2 s were considered solitary, and <2 s as part of BIRDs. ***C***, Examples of solitary IS (light blue dots) and BIRDs (dark blue dots), with BIRD durations shown as bars. ***D***, A Poincaré plot shows a sampling of inter-spike interval (ISI) pairs which can be divided into “First,” “Within,” and “Last” spikes of BIRDs or solitary spikes using the same 2 s threshold as in (***B***).

To test how hippocampal dynamics during spatial working memory are impacted by interictal activity, saline (control) or KA injected mice were implanted with drivable micro-electrodes which were positioned in the hippocampus over several days (Table 1). Once electrodes were in their final positions mice were video monitored to determine rates of seizures and interictal discharges during restful periods (total of 13.0 ± 2.0 monitoring h/mouse). As expected, mice injected with KA experienced frequent subclinical seizures (12 ± 11 of seizures/hour, Table 4), confirming that they suffer from focal TLE (Fig. 1*A*). In addition to subclinical seizures, we observed seemingly sustained interictal spiking that was categorized into two types: solitary IS and chains of spikes called brief interictal rhythmic discharges (BIRDs). Events were classified as solitary IS or BIRDs based on inter-spike intervals similar to Heining et al. (2019, Fig. 1*B*–*D*, Table 5).

**Table 4.** Rates of spontaneous seizures during monitoring

Animal ID	Number of Monitoring Sessions	Mean seizure rate (h^−1^)	Max. rate (h^−1^)	Min. rate (h^−1^)
m1	10	6.5	18.0	0.0
m2	12	5.0	15.0	0.0
m3	12	10.9	21.7	3.7
m4	14	7.2	29.1	0.0
m5	15	7.4	26.8	1.0
m6	15	11.5	24.7	1.0
m7^a^	14	36.9	50.1	6.0

Mean, maximum, and minimum seizure rates were pooled across all monitoring sessions. Seizures were defined as trains of spikes with inter-spike intervals <2 s with a train duration of at least 10 s.

^a^Animal m7 was monitored during the light-cycle, whereas all others were monitored during the dark-cycle.

**Table 5.** Rates of interictal events during monitoring sessions

Animal ID	Number of monitoring sessions	Mean interictal spike rate (Hz)	Std. dev. interictal spike rate (Hz)	Mean solitary spike rate (Hz)	Std. dev. solitary spike rate (Hz)	Mean BIRD rate (Hz)	Std. dev. BIRD rate (Hz)
m1	10	0.792	0.589	0.034	0.013	0.041	0.019
m2	12	0.227	0.139	0.020	0.006	0.030	0.012
m3	12	0.468	0.136	0.049	0.005	0.072	0.008
m4	14	0.356	0.203	0.039	0.006	0.064	0.031
m5	15	0.214	0.139	0.018	0.006	0.030	0.013
m6	15	0.266	0.067	0.033	0.009	0.043	0.008
m7^a^	14	0.708	0.136	0.038	0.007	0.069	0.008

^a^Animal m7 was monitored during the light-cycle, whereas all others were monitored during the dark-cycle.

### Epileptic mice have impaired performance on a spatial working memory task

**Figure 2.**
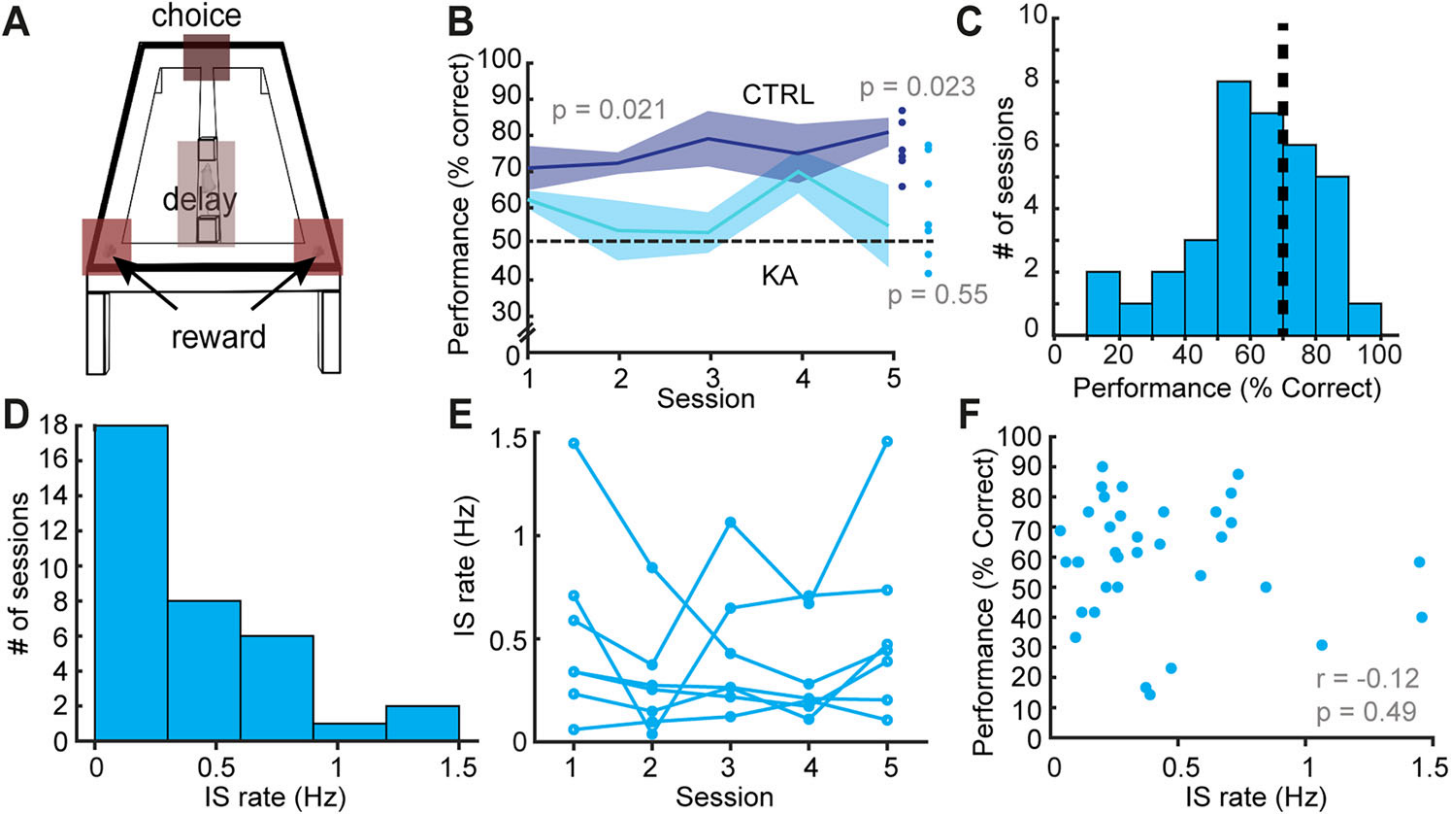
IS burden does not correlate with impaired spatial working memory performance in KA mice. ***A***, Figure 8 maze used for delayed spatial alternation with salient locations highlighted. ***B***, Control animals’ performance in the delayed alternation task (dark blue line) was significantly higher than that of KA animals (light blue). Furthermore, the mean performance across all five sessions was higher than chance (50%) only for control (CTRL) animals, shown as dots on the far right. ***C***, Day-to-day performance of KA animals was variable but interspersed with “good” sessions (>70% performance dashed line). ***D***, The mean IS rate during behavior varied across sessions. (***E***), Showing no systematic trend over time. ***F***, Finally, the IS rate burden was not found to be significantly correlated with session performance.

In addition to video-LFP-monitoring, mice were recorded during daily behavior sessions comprising a spatial working memory task flanked by rest sessions. While performing the delayed alternation spatial working memory task, mice had to alternate between visiting two sides of a Figure 8 shaped maze (Fig. 2*A*) to receive food (or liquid sucrose for m7) rewards with a 30 s delay period between trials (see Methods for training details; Hoxha and Sabariego, 2020). Over the five sessions of testing, control mice (n=6) performed significantly better than KA mice [*n* = 7; repeated measures ANOVA, *F*(1, 11)= 7.25, *p* = 0.021; Fig. 2*B*]. The difference in behavior was also observed when averaging performance across the five sessions of testing (Fig. 2*B*; control, n=6,76.4±3.1%; KA, n=7,59.34±5.2%, unpaired *t*-test, d.f. = 11, *t*-stat = 2.69, *p* = 0.021). Notably, the KA group did not perform better than the chance level of 50% correct choices (one sample *t*-test, *t*-stat = 0.63, d.f. = 6, *p* = 0.55), whereas control mice did perform significantly higher than chance level (one sample *t*-test, *t*-stat = 3.2, d.f. = 5, *p* = 0.023; Fig. 2*B*). We also noted that despite poor overall performance, the KA group exhibited individual sessions of good performance (Fig. 2*C*), suggesting that the mechanisms underlying poor performance may be dynamic.

### IS occur during working memory and their spatial distribution, but not rate, correlates with memory performance

To determine what mechanisms underlie impaired and variable memory performance in KA animals, we recorded hippocampal LFPs during task performance. Animals had high rates of IS while performing the working memory task (0.50 ± 0.07 Hz, *n* = 35 sessions = 7 animals × 5 sessions; Fig. 2*D*). While IS rates varied between sessions (Fig. 2*E*), surprisingly, the mean rate of spikes during each session was not significantly correlated with performance (Pearson *r* = −0.12, *p* = 0.49; Fig. 2*F*).

**Figure 3.**
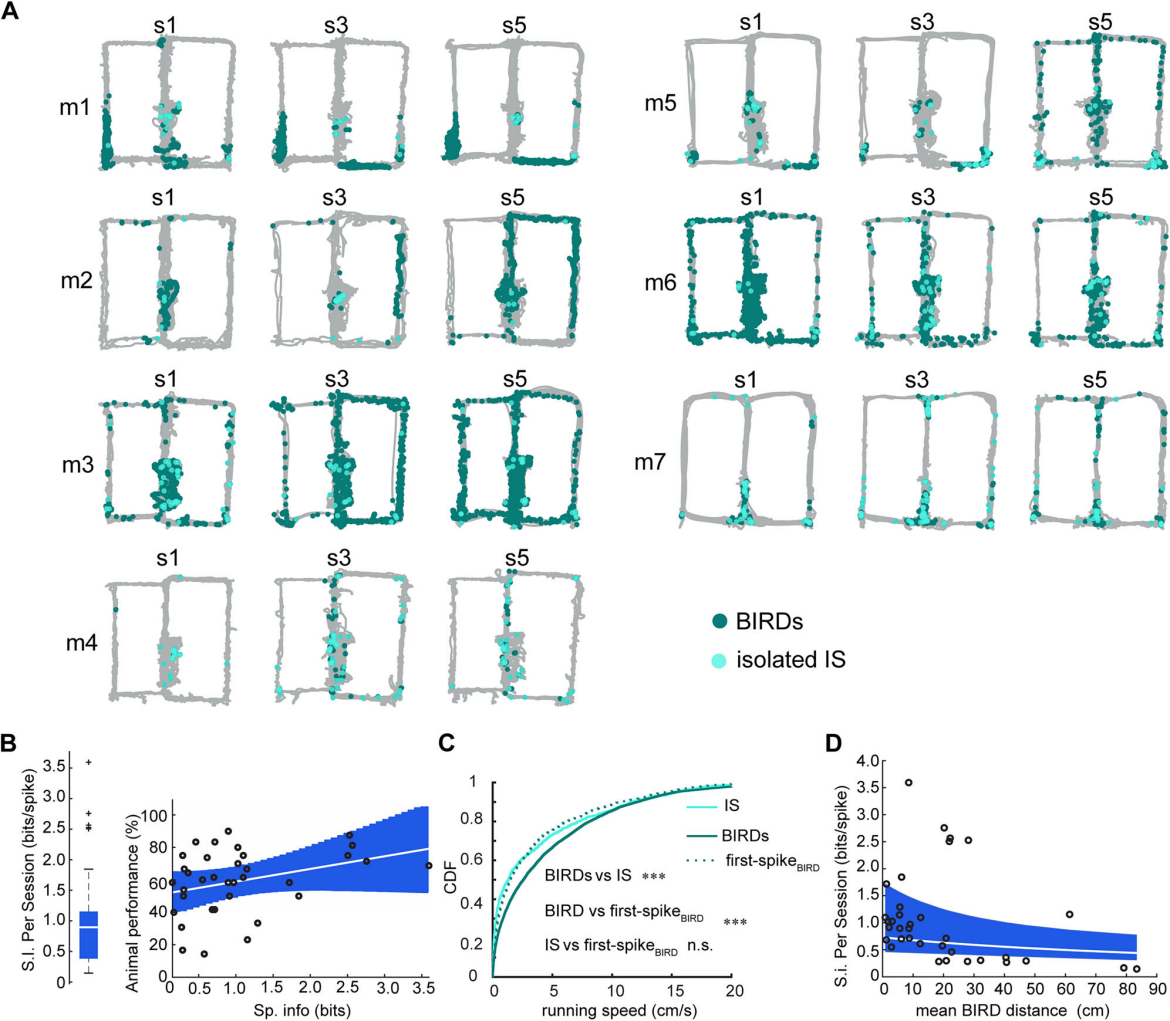
Spatial clustering of IS during working memory correlates with performance. ***A***, The locations of BIRDs (dark blue dots) and solitary IS (light blue dots) on the maze for all animals shown for three of the five sessions. ***B***, The spatial information per interictal spike was computed for each session (*N* = 35 = 7 mice × 5 sessions, left). Higher values of spatial information corresponded to a higher alternation performance predicted by a GLM (right; *p* = 0.049, see Table 6 for details). ***C***, The running speed during solitary spikes (light blue), BIRDs (dark blue), and the first spike of each BIRD (dashed) was compared to reveal that BIRDs occur at faster running speeds than solitary IS or the first spike in each BIRD. Table 7 contains statistics for the comparisons shown in ***C***, ***p < 0.001, n.s. *p* > 0.05. ***D***, Using a GLME, it was found that working memory sessions that had BIRDs associated with long running trajectories significantly explained lower values of spatial information (fixed-effect for distance term, *p*-value = 0.02, see Table 8). The marginal (unconditional) fixed-effect mean and 95% CI are shown in the blue shaded region.

Given the mean rate of spikes during the task did not correlate with behavioral performance, we examined whether the fine details of where spikes with respect to the mouse’s navigational behaviors could explain spatial memory deficits. Spikes either occurred as solitary IS (0.027 ± 0.003 Hz) or in BIRDs (0.035 ± 0.003 Hz), and were pooled to test the overall impact of all spikes on behavior. BIRDs were typically short in duration (4.5 ± 0.42 s) and comprised several spikes (14 ± 2 spikes). We noted that for some mice the spatial distribution of spikes were confined to specific areas of the maze, and were even consistent across sessions of memory testing (e.g., m1 and m7, Fig. 3*A*). Other mice exhibited patterns of spiking that extended across large portions of the maze and were more variable session to session (e.g., m3 and m6). Consistent with this observation, we found that the spatial information of IS, which is a measure of how well spiking activity predicts mouse location, was quite variable across sessions (Fig. 3*B*, left), with some sessions exceeding values of 2 bits/spike. Such high values of spatial information match those reported for individual place cells in healthy hippocampus (Skaggs et al., 1992). High spatial information of IS was weakly, but significantly correlated with a better performance on the working memory task (Fig. 3*B*, right, *p* = 0.049; see Table 6 for further statistical details).

**Table 6.** GLM coefficients comparing spatial information (SI) to animals’ per-session performance, adjusted *R*^2^ = 0.085, d.f. = 33, dispersion = 0.03

Parameter	Estimate	SE	tStat	*p*-Value
Intercept	0.5	0.05	10.0	<1 × 10^−5^
SI	0.08	0.04	2.0	0.049

To further investigate contributions to the variable nature of spatial information of interictal spiking, we calculated running speeds at the times of IS and BIRDs. Solitary IS occurred during periods of rest as reported by others (Gelinas et al., 2016), while BIRDs tended to occur when the mouse was running at faster speeds (Fig. 3*C*, see Table 7 for statistics). Interestingly the first spike in a BIRD had a speed-tuning distribution that overlapped with solitary IS (Table 7), indicating that BIRDs may initiate from quiet restful states but can encroach onto running states if the animal begins movement mid-BIRD. We reasoned that BIRDs during running would drive lower spatial information, and indeed sessions with BIRDs that spanned larger distances on the maze were correlated with lower total information per spike (Fig. 3*D*, GLME fixed-effect for distance term, *p*-value = 0.02, Table 8 for more statistics).

**Table 7.** Running speeds of IS and BIRDs

Groups	*p*-Value	*z*-Value	Rank sum
IS versus BIRDs	1 × 10^−16^	−8.3	6.6 × 10^6^
BIRD versus first spike of BIRD	9 × 10^−15^	7.8	1.3 × 10^8^
IS versus first spike of BIRD	0.07	−1.8	1.0 × 10^6^

Results of Wilcoxon rank sum test for the running speed distributions in Figure 3.

**Table 8.** Coefficient values for the gamma GLME in Equation 6, estimated dispersion was 0.08

Parameter	Estimate	SE	tStat	d.f.	*p*-Value
Intercept	1.4	0.4	3.5	33	0.001
〈*d*_*k*_〉	0.01	0.004	2.4	33	0.02
(1|animal)	0.99				

Adjusted *R*^2^ = 0.82 SE = standard error, d.f. = degrees of freedom.

### The distribution of IS in the behavioral maze is augmented in specific spatial zones of the maze

**Figure 4.**
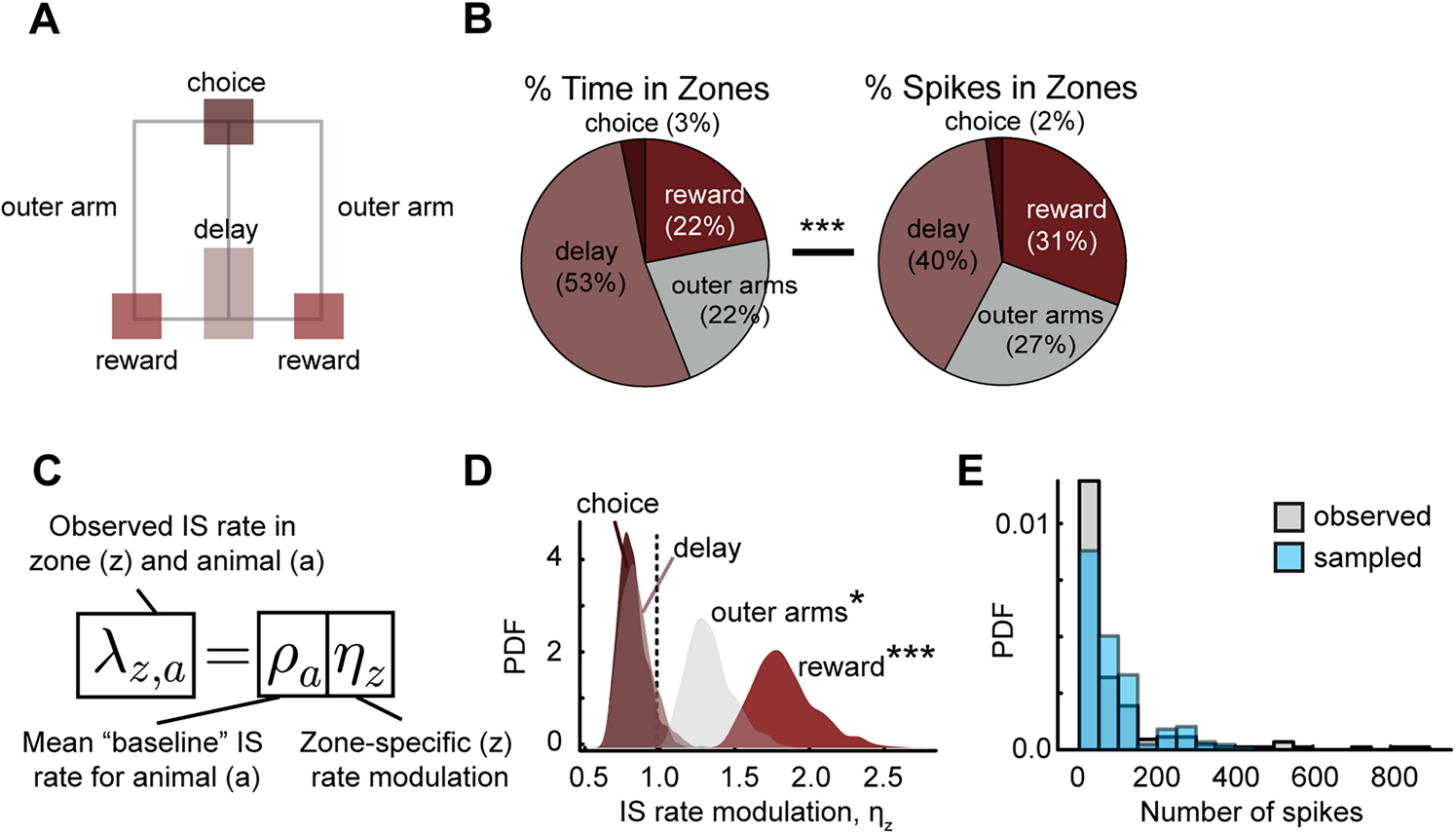
IS and BIRD rates are augmented in certain spatial zones of the maze. ***A***, For choice, delay, outer arm, and reward zones, (***B***) the proportion of time spent and the proportion of IS in each zone differed significantly from each other (*χ*^2^ test, *p*-value = 2.6 × 10^−9^, dof = 1, *χ*^2^ stat: 35.44 ). ***C***, To examine how each zone affected the IS rate of each animal, a Bayesian model was estimated (see Methods for details), where a zone-specific gain *η*_*z*_ = 1 was interpreted as a “neutral” effect. ***D***, The “outer arms” and “reward” zones had 95% highest posterior density (HPD) intervals of *η*_outer arm_ = [1.1, 1.6] and *η*_reward_ = [1.5, 2.3], respectively. ***E***, As a posterior predictive check, the distributions of IS spike counts actually observed were compared to those predicted by the Bayesian model. The bulk of the distributions (i.e., for means <400 spikes) agree whereas observed over-dispersion in the tails was not fully captured.

To see whether IS were more likely to occur at specific maze locations, we divided the maze into “Delay,” “Choice,” “Reward,” and “Outer Arm” zones and calculated both the total time each animal occupied that zone and the IS rate in that zone (Fig. 4*A,B*). The occupancy distribution was significantly different from the distribution of spikes in each zone (*χ*^2^ test, *p*-value = 2.6 × 10^−9^, dof = 1, *χ*^2^ stat: 35.44 Fig. 4*B*), indicating that the IS-generating process is non-stationary. To understand the zone-specific effects on the IS rate, we modeled the non-stationarity as a non-homogeneous Poisson process in which a “baseline” spike rate, *ρ*_*a*_, which is specific to each animal, is scaled by zone-specific gain factor, *η*_*z*_, unique to each zone but shared between all animals (Fig. 4*C*).

The posterior distributions of *η*_*z*_ in each zone were compared to a null value of 1 indicating the absence of a zone-specific modulatory effect on the IS rate. The “Reward” zone’s gain significantly deviated from 1 (Fig. 4*D*; *η*_reward_ = [1.5, 2.3], 1 − *α* = 95% HPD interval, *N* = 7 animals × 5 sessions × 4 zones) and the “Outer Arm” zones’ term also deviated from 1 (*η*_outer arm_ = [1.1, 1.6] HPD interval). In other words, the IS rate was significantly elevated from baseline when the animal occupied reward zones and when the animal ran down outer arms to the reward zones, but the IS rate was consistent with baseline at all remaining locations on the maze. These results are consistent with our findings regarding spatial information, as we would expect that sessions with IS augmented at reward sites would have high spatial information, whereas sessions that had IS while the animal ran down outer arms of the maze would drive lower spatial information.

The model’s fit and inferences were inspected to assess model plausibility. We validated the model’s inferences by confirming that the distribution of posterior means of *ρ*_*a*_ [0.46 ± 0.23 Hz, *n* = 7 mice, mean ± 95% confidence interval (CI)] agreed with the “naïve” time averaged IS rate (0.50 ± 0.07 Hz, *n* = 7 mice × 5 sessions), which was not explicitly given as data to the model. The mean values predicted by the model were compared directly to the observed data, where it was found the model distribution qualitatively agreed with the observed data (Fig. 4*E*).

### Reward zone LFP discriminability predicts animals’ working memory performance

**Figure 5.**
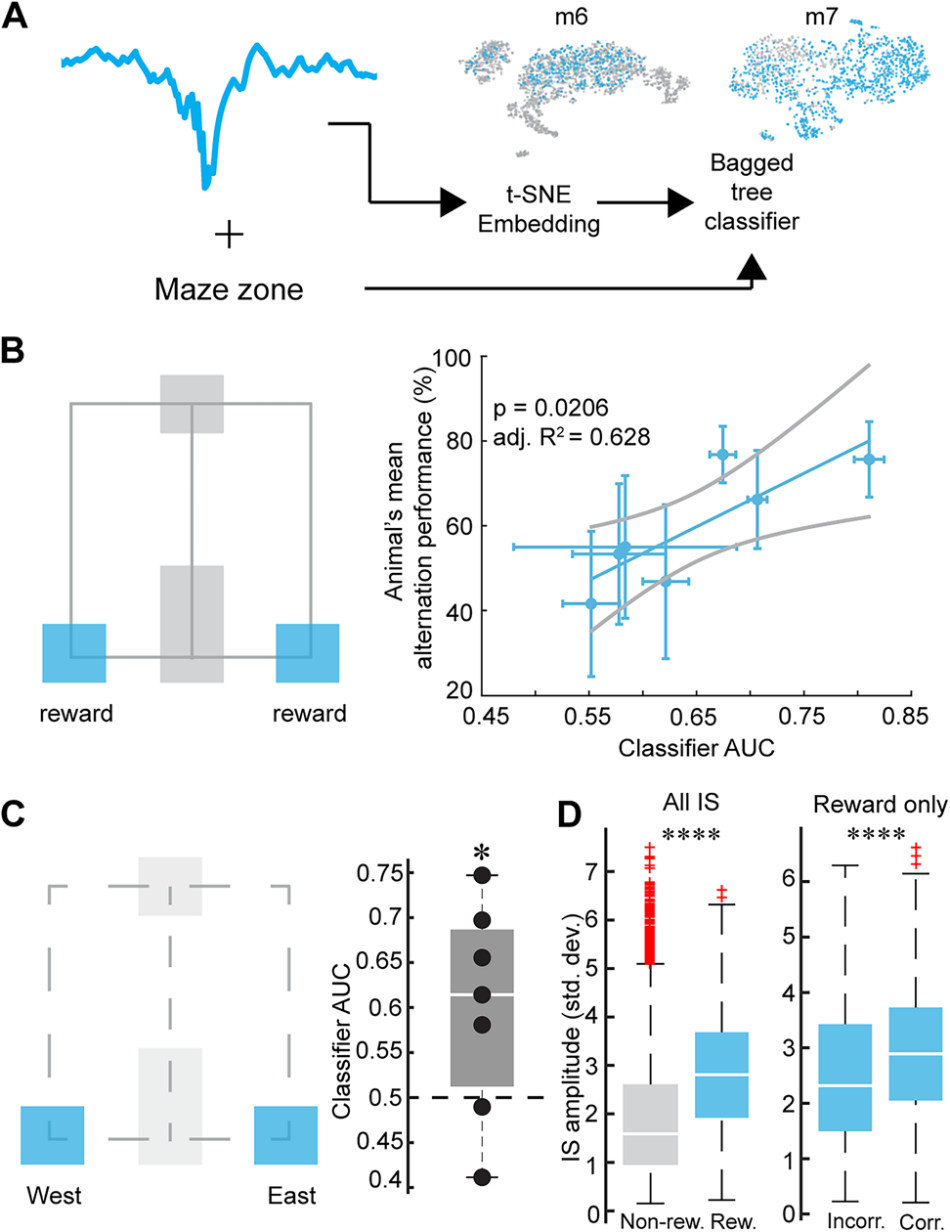
The ability to decode reward zones from the IS LFP predicts animals’ memory performance. ***A***, The normalized LFP from each IS was non-linearly embedded into a two-dimensional space using the *t*-SNE algorithm (Van der Maaten and Hinton, 2008). A bagged ensemble of trees binary classifier (Breiman, 1996, 2001) was trained on the embedded IS LFP to decode whether the IS happened within a reward zone (blue) or not (gray dots). The *t*-SNE embedding of two representative animals’ LFP are shown. ***B***, The classification was evaluated using the receiver operating characteristic (ROC) area-under-the-curve (AUC). The AUC value was associated with the animal’s mean performance across the five sessions of behavior (*p* = 0.0206, see Table 9 for further details). Solid line is the mean and light lines are 95% CI of the regression model, error bars show 95% CI of individual data points. ***C***, The analysis was repeated for IS generated in reward zones only to see if animals maintained a representation of east versus west reward zones. The population mean of the AUC values was significantly greater than 0.5 chance level (tStat = 2.24, df = 6, sd = 0.12, *p* = 0.033, one-sided *t*-test; *p < 0.05). ***D***, The root-mean-squared amplitude, normalized by the standard deviation per given animal, was computed for each IS waveform. The left panel shows IS at reward zones was larger than all other zones (tStat =−40, df = 16,299, sd = 1.3, two-sided *t*-test; ****p < 0.001). The right shows for spikes in reward zones only, IS during which the animal was rewarded were slightly larger (tStat =−8.2, df = 4,721, sd = 1.2, *p* = <0.001, two-sided *t*-test).

Given the significantly elevated IS rate in reward zones (Fig. 4*D*), which in some animals exhibited place cell like precision across sessions (Fig. 3), we hypothesized that the IS LFP at reward zones may contain latent information regarding the location of the animal on the maze. Several studies have shown that features of the hippocampal LFP signal can be decoded to reveal a continuum of generating mechanisms (Navas-Olive et al., 2020, 2022, 2023; Sebastian et al., 2023, 2024), and even into variables describing the animal’s behavioral state including position (Agarwal et al., 2014; Cao et al., 2021; Douchamps et al., 2024) and social context (Mohapatra et al., 2024). After non-linearly embedding each IS LFP into a two-dimensional space (Fig. 5*A*), a bagged ensemble of trees binary classifier (Breiman, 1996), sometimes referred to as a “random forest” (Breiman, 2001), was trained to discriminate between IS which occurred at reward sites versus those that did not. The classifier’s performance as measured by the ROC (Fig. S1; AUC), was able to predict the animal’s mean performance on the alternation task (Fig. 5*B*, Table 9). Furthermore, when considering a classifier on only spikes that occurred in the reward zones, east and west reward sites could also be discriminated above chance level (Fig. 5*C*). This suggests that mice which generate IS in reward zones that are sufficiently distinct from IS in other locations on the maze have better spatial working memory, and that reward-IS carry spatial signals that are informative and potentially could be useful for solving the task. This is consistent with reports that SWR in healthy animals recruit cells which encode locations near rewarded locations (Singer and Frank, 2009; Joo and Frank, 2018). The effect of classifier AUC on predicting animals’ performance was consistent when controlling for mean spatial information, suggesting that both discriminability of reward-related IS and spatial information of IS are important and explain different aspects of the variance (Table S1). Interestingly, IS which occurred in reward zones had significantly larger relative amplitudes than those that occurred in other locations on the maze (Fig. 5*D*). Similarly, when considering only IS within reward zones, the relative amplitudes for those which occurred during correct choices were also significantly larger than those during incorrect choices (Fig. 5*D*). This is consistent with reports that SWR in healthy animals at reward sites are larger in amplitude and longer in duration than at unrewarded locations (Singer and Frank, 2009). Thus, the reward-related changes in IS features we have observed mirror those of reward SWR, suggesting that the decodability and amplitude differences we observe in IS may be driven by similar mechanisms that also recruit larger SWR and engage ensembles that encode locations near rewards.

**Table 9.** Coefficient values for the regression model in Figure 5*B*

Parameter	Estimate	SE	tStat	d.f.	*p*-Value
Intercept	−22.2	24.6	−0.9	5	0.41
〈AUC〉	126.1	37.8	3.3	5	0.02

Adjusted *R*^2^ = 0.628 SE = standard error, d.f. = degrees of freedom. Comparison against constant model: F statistic = 11.1, *p* = 0.0206.

### Task engagement state is related to performance and IS rates during the delay phase

A key phase of working memory is the delay phase. In our case, this corresponds to the 30 s period between trials when animals must maintain representations of the past to inform future decisions or “hold on” to a future plan. In healthy animals, it is known that SWR during delay phases often replay locations of recently visited reward locations (Gillespie et al., 2021), which is thought to support future decisions to not revisit that location on the next trial. Furthermore, interrupting SWR in between components comprising a multi-step task selectively impairs memory performance (Jadhav et al., 2012), suggesting that SWR are critical for memory processes which take place on similar timescales as behavior. We therefore were interested in IS in the delay phase and whether or not delay phase IS could play a similar role to that of delay phase SWR.

**Figure 6.**
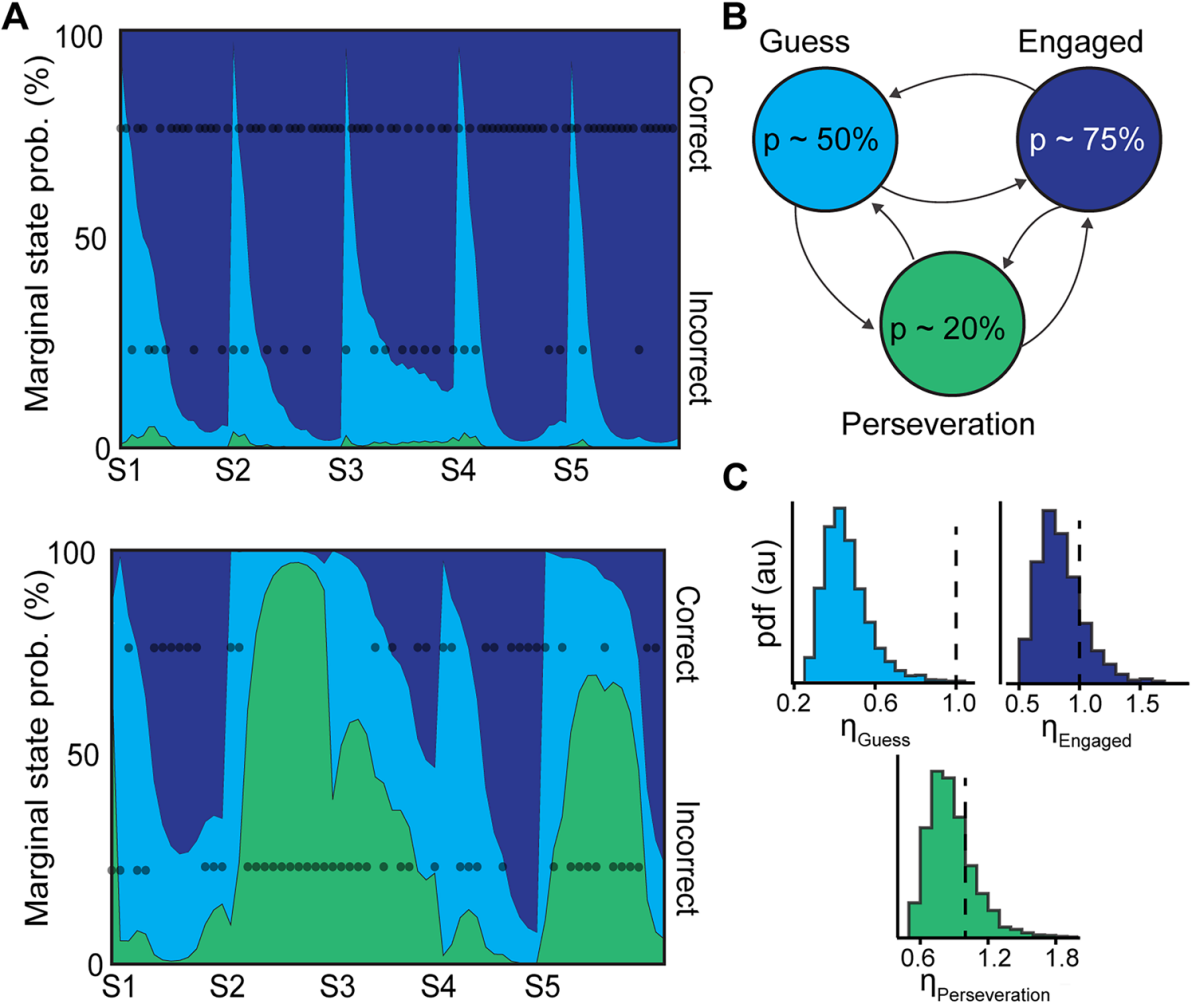
Mice switch between distinct behavioral states with differing IS rates from trial-to-trial. ***A***, The performance of two example animals are shown. The shaded areas represent the estimated marginal probability of being in one of three states (***B***) determined using a hidden Markov model. The trial-to-trial performance was partitioned into three states. The dots show the outcome (correct vs incorrect) for each trial. ***B***, The probability of a correct alternation followed a Bernoulli random variable (rounded here for simplicity of interpretation). ***C***, In the state labeled as “Guess,” the rate of IS in the delay zones was down-modulated by a gain term *η*_Guess_ = [0.27, 0.65], indicating a 95% HPD excluding unity. The other gain terms were consistent with unity, i.e., a neutral effect on the baseline rate.

First, we accounted for variations in engagement with the memory task which may co-vary with IS rates. Task engagement is known to fluctuate in healthy animals between distinct states with different error rates (Ashwood et al., 2022). Therefore, we first estimated distinct task engagement states. Using the mice’s trial-to-trial performance, we inferred three discrete task-related behavioral states corresponding to low [p(Correct) = 19%], medium (53%), and high (75%) success rates using a HMM scheme (Fig. 6). Naturally, the medium level state is consistent with a random guess, and the high-level engaged state corresponds to performing the task correctly with few errors. The low performance state is consistent with the strategy of perseveration, i.e., choosing the last visited site repeatedly. Within a single day, the mice typically transitioned from an initial “guessing” state to an “engaged” state, or relatively less often a “perseveration” state characterized by many errors in a row (Fig. 6*A*). Control animals’ performance also was represented with an HMM (Fig. S2) of similar structure to that in Figure 6*B*. Importantly, the probability of remaining in a perseveration state was lower in controls than in epileptic mice (Fig. S3). In agreement with computational and psycho-physical investigations of reaction time and decision certainty (Beck et al., 2008; Churchland et al., 2008; Kiani et al., 2014), mean time to exit the delay zone was inversely related to the probability of correct choice as summarized in Table 10.

**Table 10.** The mean delay exit time (time to exit delay zone after 30 s interval elapsed) was estimated from 1,000 samples drawn using the hierarchical bootstrap method (Saravanan et al., 2020) for each discrete state estimated by the Viterbi algorithm

State (% corr.)	Mean delay exit time (s, 95% CI)
Perseveration (19%)	[11.9, 12.2]
Guess (53%)	[7.8, 7.9]
Engaged (75%)	[7.55, 7.6]

To understand the relationship between the inferred task-related behavioral state and IS, we examined whether the rate of IS in the delay zone on each trial was different in each state. We found that the distributions of the rate of delay zone IS in each behavioral state, estimated by the Viterbi algorithm, did not share a common center location, suggesting that the rate of IS in the delay zone is related to behavioral performance (Kruskal–Wallis rank sum test *χ*^2^ approximation, *p*-value <0.0001, *χ*^2^ = 19.4, dof = 2). To estimate the magnitude of state-specific effects on IS rate, a firing rate model similar to the maze zone analysis (Fig. 4) was built to infer a “baseline” IS rate only in the delay zone for each animal, *ρ*_*a*_ (0.51 ± 0.36 Hz, *n* = 7 mice, mean ± 95% CI of posterior means; Fig. 6*C*). With the interpretation of a gain of 1 being a neutral effect, the model predicts that the “guess” state (*η*_Guess_ = [0.27, 0.65], 95% HPD credible interval, *N* = 504 = 7 animals × 5 sessions × M trials/day, where M is different for each animal on each day) was associated with a significant reduction in delay zone IS, while both perseveration (*η*_Perseveration_ = [0.51, 1.26]) and engagement (*η*_Engaged_ = [0.51, 1.22]) IS rates were not modulated and were thus relatively high (Fig. 6). Given the bimodal effect of high rates of IS during the delay phase on behavioral performance (i.e., high rates associated with strong and poor performance), we turned to a modeling approach to investigate potential replay content during IS.

### A simple model of IS and hippocampal place coding

**Figure 7.**
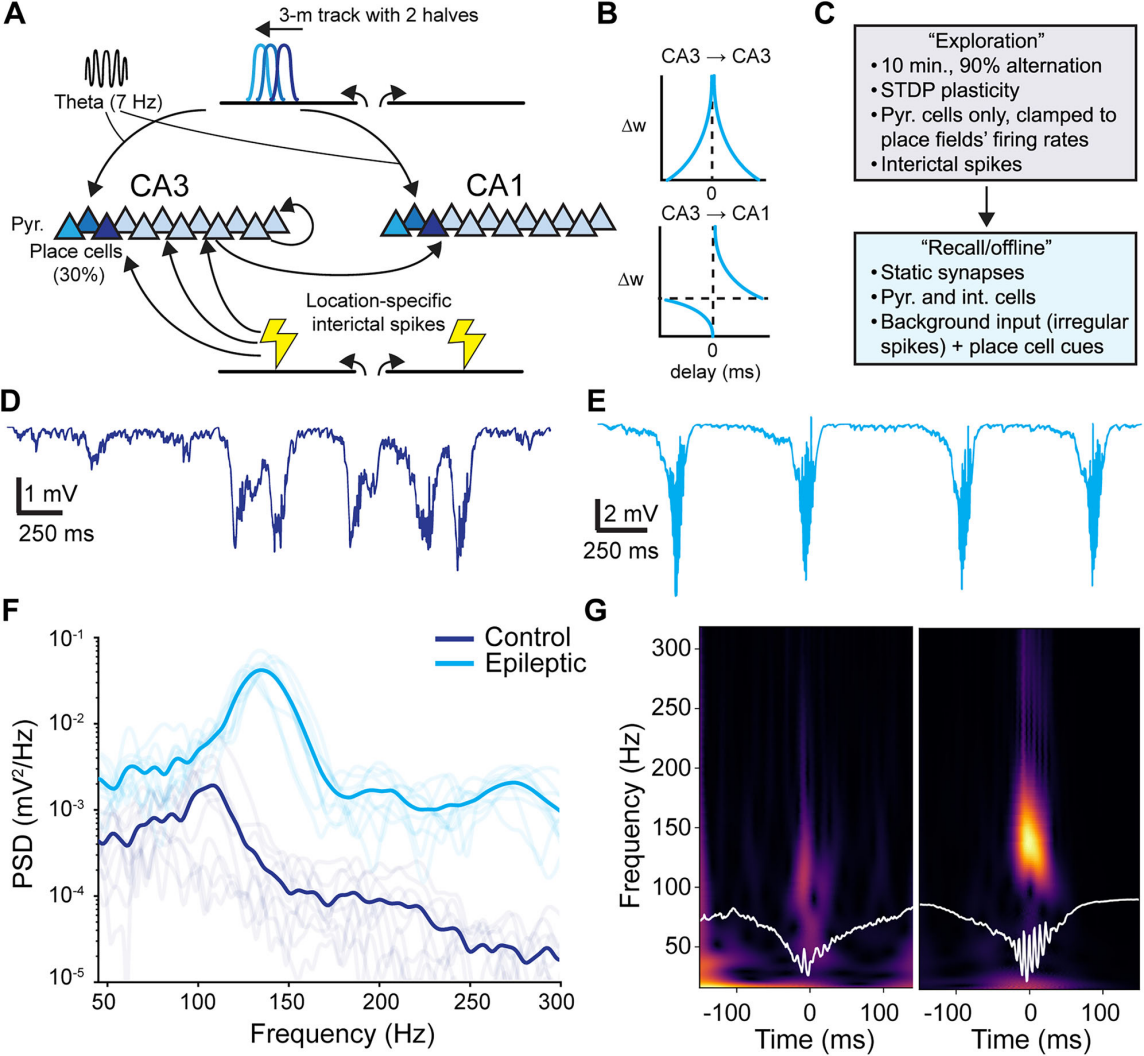
Providing interictal spikes (IS)-like input to a model of hippocampal place coding induces IS with spontaneous high frequency oscillations (HFOs). ***A–C***, A schematic of the “exploration” phase of the spiking model. The virtual track was divided into two halves and IS were delivered at two locations shown schematically by the lightning bolts. ***B***, The STDP weight updating rules for pyramidal cell synapses during exploration are shown schematically. Representative simulated LFP traces generated from a (***D***) control and (***E***) epileptic network are shown with scale bars (250 ms and 1 or 2 mV, respectively). ***F***, Ten replica networks were created and the resulting spectrograms of their spontaneous replay-like bursts are shown with the group means in dark lines. ***G***, The mean replay oscillations from one replica network are shown along with their continuous wavelet transforms.

Replays during SWR (Pastalkova et al., 2008; Malvache et al., 2016) are thought to be important for prospective planning and consolidation of recent actions (Dragoi and Tonegawa, 2011; Drieu et al., 2018; Mattar and Daw, 2018; Gillespie et al., 2021). We sought to assess the plausibility that IS during behavior (at reward and on outer arms) interfere with mechanisms of spatial memory, especially in regards to replay events during offline states and intertrial periods (i.e., during the delay phase). Therefore, we built an idealized model of CA3 and CA1 place coding. We modified an existing model of place coding induced by STDP (Ecker et al., 2022; Liu et al., 2023; Liao et al., 2024) to include IS which were simulated by delivering bursts of spikes to CA3 pyramidal cells (Fig. 7*A*–*C*). A single burst was delivered per trial in the same relative location in the track. In the model, a mouse “explores” a linear track where it can go left or right with 90% chance of picking the opposite of the last trial (Fig. 7*C*) and is “teleported” back to the center of the maze to begin the next trial. After training with STDP, the spontaneous network activity was then studied to get a general sense of HFO dynamics in the epileptic network. Networks that received interictal-like pulses on the maze produced larger amplitude and higher frequency HFOs compared to control networks (Fig. 7*D*–*G*). Simply by including interictal-like stimuli during training, the network spontaneously generated population events that recapitulated the major qualitative differences observed in the LFPs of ripples and pathological HFOs (Ewell et al., 2019).

We then studied the spiking content of spontaneously generated replay events in the model. Like previous reports (Ecker et al., 2022; Liu et al., 2023), we observed spontaneous “replay” events of place cells in the offline state in control and epileptic networks (Supp. Fig. S4*A*). We used the population vector approach (Zhang et al., 1998) to reconstruct the maze positions represented by the network activity during each replay event and in each simulated subfield (Fig. S4*B*). Like in previous reports using similar models (Ecker et al., 2022; Liu et al., 2023), such replays were generally longer lasting and involved longer trajectories than are observed in real data, but nonetheless give a lens for comparing between control and epileptic networks.

### Spatial distribution of IS during simulated online exploration affects the quality of offline replay events

**Figure 8.**
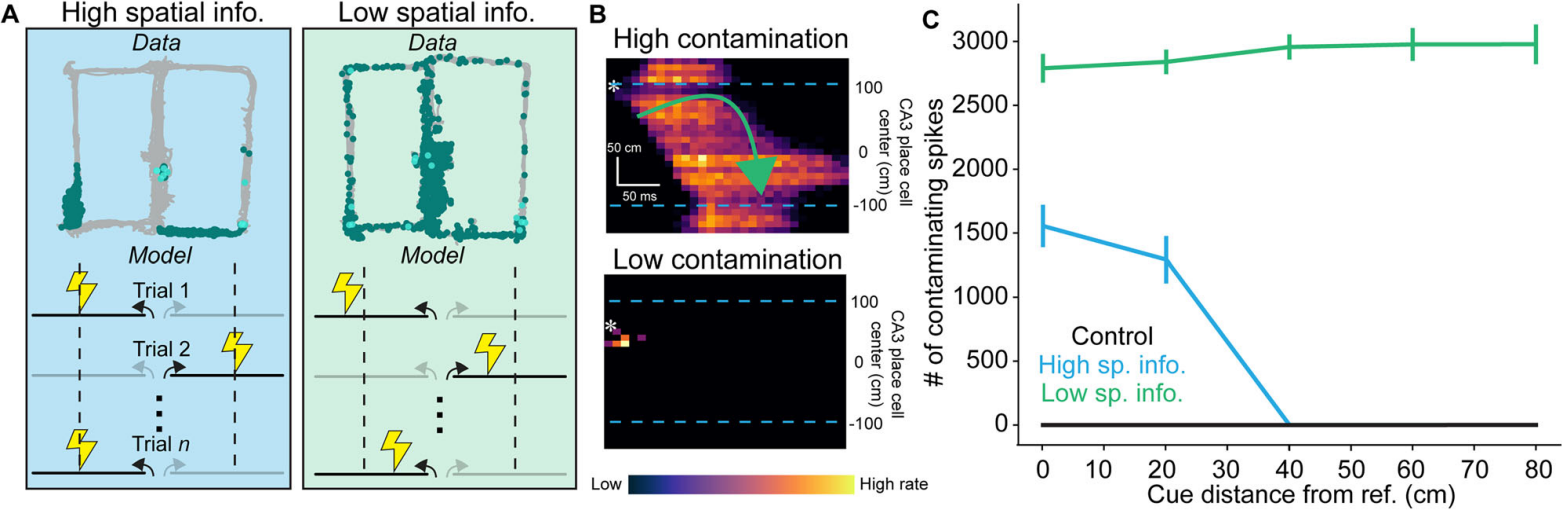
Spatial spread of IS leads to impaired replay in a hippocampus network model. ***A***, In the IS data in vivo, we found two broad regimes: one where IS were restricted in space (high spatial info, blue), and one where IS were spread across the maze (low spatial info, green). To assess whether these disparate spatial statistics would differentially affect the hippocampal network, we trained the network in Figure 7 on two different cases: one where on each trial, the IS (lightning bolt) was delivered to place cells at the same reference point on each arm (dashed line), and another where the IS was distributed uniformly in space. These cases were referred to as high and low spatial information simulations, respectively. A control network was also created, where no IS were delivered. ***B***, Representative examples of cued replays which led to high and low contamination replays, respectively. “Contamination” refers to the spread of the replay onto the un-cued arm. The trajectory of the contaminated replay is highlighted by the green curved arrow. The asterisks show the location of the place fields cued, the blue dashed lines show the location of the dashed lines in panel ***A***. ***C***, The number of contaminating spikes was low and constant in the control network, and was independent of the distance between the cued location and the reference point. In the high spatial information network, significantly worse contamination emerged for cues near to the reference point (<30 cm), and was reduced to near control levels for cues further from the reference point. For low spatial information networks, replay was contaminated above control levels at all cue distances. Error bars show one standard error of the mean.

Our in vivo experiments showed significant variability in the distributions of IS on the maze (Fig. 3*A*,*B*). We were interested in how the spatial distribution of IS during exploration of the maze impacted the content of replay of remote locations (like reward locations). We simulated “cued” replay by stimulating a subset of place cells with a brief pulse of activity to induce a population event (Ecker et al., 2022). Such cued replays were performed in networks that were trained in two cases that reflected the two extreme patterns of IS distributions we observed in our real data (Fig. 8*A*): the first regime (high spatial information) where simulated IS were delivered to place cells with fields at the same relative locations on the virtual maze and a second regime (low spatial information) where the location of each IS was varied randomly from trial-to-trial. We considered the location of the IS in the high information case as a “reference point.” Then, the relative spread of replay content beyond the cued arm was measured as the number of spikes in the un-cued arm of the maze. This metric served as a proxy of replay “contamination” or over-generalization (Fig. 8*B*). This was measured as a function of distance between the place fields corresponding to cued cells and the reference point (Fig. 8*A*,*C*). In the high spatial information case, over-generalization of the replay beyond the cued zone was restricted to cue distances <30 cm from the reference point. In the low spatial information case, over-generalization occurred at all cue distances from the reference point. In the <30 cm region, the level of contamination between high and low spatial information were both elevated (Fig. 8*C*). In contrast to both conditions, control networks had low levels of contamination regardless of the distance to the reference point (Fig. 8*C*). These results suggest that when IS are scattered across the maze, the network is unable to generate precise replays during offline states and potentially during the delay phase of the working memory task. For example, an animal with broadly distributed spikes would not be able to replay previously visited reward locations (or any other locations on the maze) in isolation. In contrast, an animal with IS restricted to just the rewarded areas could still achieve replays with contamination levels comparable to controls but only for maze locations far from reward. Our model predicts corruption of replay could partially explain the low performance we observed in animals that had IS with low spatial information.

## Discussion

### IS correlate with impaired spatial memory in a spatially specific manner

Our recordings in freely behaving epileptic mice reveal that IS occurred frequently during a hippocampal dependent spatial working memory task (Figs. 1 and 2). While the average rate of spikes during the task did not correlate with memory impairments (Fig. 2), the spatial specificity of IS did. IS rates were augmented during the active encoding phases of the task and either occurred clustered, proximal to reward sites or dispersed on maze arms (Fig. 3). Spatially dispersed IS that carried low spatial information correlated with poor performance. In part, the dispersed pattern of IS could be explained by the fact that BIRDs were sustained during locomotion (Fig. 3*C*,*D*) and thus “smeared” IS across the maze. On the other hand, when IS were clustered near reward sites, they were spatially informative and were distinct in LFP shape from other IS on the maze and between reward locations (Fig. 5). Thus IS at reward sites may be reliably engaging ensembles with spatial information, supporting better memory performance.

First, considering the case where IS were spread across the maze, our observations raise the possibility that altered rates and phase-of-firing of inhibitory neurons during theta states (Lopez-Pigozzi et al., 2016; Shuman et al., 2020) allow IS to transiently break through even during locomotion. Similarly, others have observed that dis-inhibiting CA3 enables the generation of IS-like events during theta (Boehringer et al., 2017; McHugh, 2023). Another possibility is that cholinergic drive, which is typically high during running and is known to inhibit population synchrony (Vandecasteele et al., 2014) might be reduced in epilepsy. We hypothesize that place cell recruitment during spatially non-specific IS are a mechanism that contribute to the observed reduction in place field specificity and stability in epileptic mice reported in several studies (Liu et al., 2003; Zhou et al., 2007; Karnam et al., 2009; Titiz et al., 2014; Ewell et al., 2019; Sakkaki et al., 2020; Shuman et al., 2020).

We also observed cases when IS were restricted to reward sites and exhibited reward-related changes, which taps into an interesting line of investigation between reward and replay-based memory mechanisms. We find a suite of reward-related changes in IS that mirror those which have been reported for SWR in health (Singer and Frank, 2009; Ambrose et al., 2016; Joo and Frank, 2018; Gillespie et al., 2021). For example, we observed that the amplitude of IS were larger in rewarded contexts (Fig. 5*D*) which could be linked to mechanisms that drive increases in the number of pyramidal cells recruited to SWR in rewarded contexts in healthy animals (Singer and Frank, 2009). Finally, we found that the ability to discriminate IS LFP waveforms triggered in reward zones from those triggered in other locations predicted memory performance (Fig. 5*B*,*C*), which to our knowledge has no analog for SWR that has been reported. Thinking more broadly, there is a connection between reward-related neuromodulation and epilepsy in general (Starr, 1996; Haut and Albin, 2008). In slice experiments, adding dopamine agonists increases the rate of epileptiform bursting and propagation distance (Suppes et al., 1985; Bandyopadhyay et al., 2005; Goda et al., 2008), and dopamine and serotonin receptors are a potential target for anticonvulsant drugs (Clinckers et al., 2004; Hablitz, 2004; Goda et al., 2008). Future work should focus on dissecting whether activity in the dopamine system can explain the reward-related changes in IS rate and waveform changes we have observed.

### IS may both aid and interfere with memory-based planning remote replay

Our modeling results suggest that errors made in different behavioral states can be explained by the content replayed in the hippocampus during remote replay which would likely occur during the delay period of our working memory task. While we do not directly observe the spiking content replayed in each IS in vivo, the decoding analysis in Figure 5 suggests that working memory may depend on generating delay and choice zone IS which have features that are distinct from those of reward zone IS (suggestive of engagement of distinct neuronal ensembles). Furthermore, the cueing simulations in Figure 8 suggest a biologically plausible mechanism for error generation.

We found both engagement and perseveration are associated with baseline interictal activity during the delay period (Fig. 6). In conjunction with our modeling results, we speculate that IS during the delay phase are mimicking delay phase SWR replay dynamics, but that the content of replay is either helpful (engagement) or harmful (perseveration). In contrast, when there is no information available, reflected by suppressed IS rates during delay, we speculate that the animal resorts to guessing. In the case of perseveration, spread of replay content to un-cued areas during IS in the delay zone could lead to the mouse repeatedly visiting the last visited reward area due to a failure to form a cognitive representation of state transitions needed to complete the task efficiently (Igata et al., 2021; Garvert et al., 2023). However, if the replay during an IS remains contained to the cued area (perhaps in the Engaged state, or if the animal has “high information” IS spatial distribution as like in Fig. 8) this could enable the ability to make optimal plans (Mattar and Daw, 2018; Jensen et al., 2024) or to maintain an accurate cognitive map for alternation behavior (Gillespie et al., 2021), or a mixture thereof (Ólafsdóttir et al., 2018; Diekmann and Cheng, 2023). In other words, the IS-induced replay could serve a role analogous to SWR-mediated replay under certain conditions but can also generate completely pathological activity depending on the pattern of IS elsewhere on the maze.

### Limitations and future directions

Our study makes several predictions about the impact of IS on working memory that can be directly tested with future single unit studies. As mentioned above, several studies in rodents with TLE have revealed disruption of single-cell properties exhibited during theta states such as reductions in place field specificity and stability (Liu et al., 2003; Zhou et al., 2007; Karnam et al., 2009; Titiz et al., 2014; Ewell et al., 2019; Sakkaki et al., 2020; Shuman et al., 2020), contamination of phase-of-firing relationships to underlying theta and gamma oscillations (Lenck-Santini and Holmes, 2008; Barry et al., 2016; Lopez-Pigozzi et al., 2016; Sakkaki et al., 2020; Shuman et al., 2020), and aberrant post-ictal remapping (Zhou et al., 2007). We predict that IS, especially those that encroach on theta states (Chauviere et al., 2009; Ge et al., 2017; Fu et al., 2018), contribute to the development of such single-cell pathology. Additionally, based on our simulations, we predict that IS could contribute to problems with hippocampal replay such that replay is over-generalized when IS are unrestricted during theta states.

Our simulations based on Ecker et al. (2022) include only STDP. However, emerging evidence has implicated a non-Hebbian form of plasticity called behavioral time-scale plasticity (BTSP) in the formation, updating, and remodeling of hippocampal place cell representations (Bittner et al., 2017; Milstein et al., 2021; Grienberger and Magee, 2022; Vaidya et al., 2025). Recent computational models (Madar et al., 2025; Vaidya et al., 2025; Wu and Maass, 2025) have provided frameworks to simulate BTSP, however we chose not to include BTSP mechanisms in our simulations because there are important unanswered questions regarding the physiology underlying BTSP in epilepsy. For example, there are changes to dendritic calcium dynamics in epilepsy (Masala et al., 2023) and death of EC layer 3 projections to CA1 (Du et al., 1993, 1995; Schwarcz et al., 2000; Feng et al., 2025). Given these changes to elements of the BTSP circuit in epilepsy, it is difficult to model, but it is worth noting that interpretations from our simulations may be limited.

A key prediction of this study is that IS may not always be negative for memory processing. In fact, we predict that at times IS seem to functionally replace SWR (i.e., at reward sites, and sometimes during delay periods). Such complexity indicates that future studies aimed at targeting IS to ameliorate memory deficits will need to be “smart.” For example, studies employing optogenetic blockade of all IS, versus selective blockage of those deemed more pathological will be essential to determine the proper course of therapeutic intervention.

## Data Availability

All code and data needed to reproduce the findings is available for free as a Github repository (EwellNeuroLab: InterictalSpikes-Behavior). Model code is available on Github (justidy1: IctalPlaceCell). Raw recordings will be made available upon reasonable request.
